# The complete genome sequences of poxviruses isolated from a penguin and a pigeon in South Africa and comparison to other sequenced avipoxviruses

**DOI:** 10.1186/1471-2164-15-463

**Published:** 2014-06-12

**Authors:** Kristy Offerman, Olivia Carulei, Anelda Philine van der Walt, Nicola Douglass, Anna-Lise Williamson

**Affiliations:** 1 Division of Medical Virology, Department of Clinical Laboratory Sciences, University of Cape Town, Cape Town, South Africa; 2 Central Analytical Facility, DNA Sequencer, Stellenbosch University, Stellenbosch, South Africa; 3 Institute of Infectious Disease and Molecular Medicine, Faculty of Health Sciences, University of Cape Town, 7925 Cape Town, Observatory, South Africa; 4 National Health Laboratory Service, Groote Schuur Hospital, Cape Town, South Africa

**Keywords:** Avipoxvirus, Poxvirus, Pigeonpox, Penguinpox, Genome, Vectors

## Abstract

**Background:**

Two novel avipoxviruses from South Africa have been sequenced, one from a Feral Pigeon (*Columba livia*) (FeP2) and the other from an African penguin (*Spheniscus demersus*) (PEPV). We present a purpose-designed bioinformatics pipeline for analysis of next generation sequence data of avian poxviruses and compare the different avipoxviruses sequenced to date with specific emphasis on their evolution and gene content.

**Results:**

The FeP2 (282 kbp) and PEPV (306 kbp) genomes encode 271 and 284 open reading frames respectively and are more closely related to one another (94.4%) than to either fowlpox virus (FWPV) (85.3% and 84.0% respectively) or Canarypox virus (CNPV) (62.0% and 63.4% respectively). Overall, FeP2, PEPV and FWPV have syntenic gene arrangements; however, major differences exist throughout their genomes. The most striking difference between FeP2 and the FWPV-like avipoxviruses is a large deletion of ~16 kbp from the central region of the genome of FeP2 deleting a cc-chemokine-like gene, two Variola virus B22R orthologues, an N1R/p28-like gene and a V-type Ig domain family gene. FeP2 and PEPV both encode orthologues of vaccinia virus C7L and Interleukin 10. PEPV contains a 77 amino acid long orthologue of Ubiquitin sharing 97% amino acid identity to human ubiquitin.

**Conclusions:**

The genome sequences of FeP2 and PEPV have greatly added to the limited repository of genomic information available for the Avipoxvirus genus. In the comparison of FeP2 and PEPV to existing sequences, FWPV and CNPV, we have established insights into African avipoxvirus evolution. Our data supports the independent evolution of these South African avipoxviruses from a common ancestral virus to FWPV and CNPV.

## Background

Avipoxviruses have been shown to naturally infect 232 of the approximately 9,000 described species of both wild and domestic birds [1], yet relatively little is known about the host range and genetic diversity of these viruses. There are currently only ten defined- and three tentative avipoxvirus species [2] with species names assigned according to the bird species which they infect or from which they were isolated [1]. This current method of taxonomy and classification has been criticized because it has been found that multiple avian species can be infected by a single viral species and/or multiple viral species can infect a single avian species [3-6]. Phylogenetic relationships of avipoxviruses have been analyzed based on the gene corresponding to vaccinia virus (VACV) P4b (*fpv*167, VACV A3L) [3-8], indicating that all avipoxvirus strains cluster into 3 major clades, namely, A (Fowlpox (FWPV)-like), B (Canarypox (CNPV)-like) and C (Psittacine). Clade A can be further divided into seven subclades (A1-A7) and Clade B is comprised of three subclades (B1-B3) [6].

Thus far, the genomes of only three avipoxviruses have been published; a pathogenic American strain of fowlpox virus (FPVUS) [9], an attenuated European strain of fowlpox virus derived from European FWPV HP1 passaged over 400 times in chick embryo fibroblasts (FP9) [10], and a virulent canarypox virus isolated from a canary (CNPVATCC VR-111) [11]. Comparison of the CNPV and FWPV genomes reveals overall synteny in genome arrangement with similar genetic complements. They do however, exhibit significant differences in the terminal, variable regions as well as in three internal, variable regions which is in contrast to the conservation of central genomic regions in other Chordopoxviruses (ChPVs). These variable regions within the conserved central region of the genomes occur near the junctions of areas that were identified in FWPV as rearranged relative to other ChPVs and contain genes involved in virus-host interactions [9,11]. Avipoxviruses are considerably divergent from other ChPVs [9,11] and may constitute a separate subfamily within the *Poxviridae* family [11-13].

FP9, FPVUS and CNPV have large genomes of 258, 280 and 365 kbp encoding 238, 260 and 320 open reading frames (ORFs) respectively [9-11]. 90 genes have been found to be conserved amongst all ChPVs and to comprise the minimum essential genome [14]. The remainder of the genetic component of avian poxviruses is largely made up of immunomodulatory and host specific genes located in the terminal regions of the genome, that have allowed the viruses to take advantage of their unique hosts.

Laidlaw and Skinner [10] compared virulent (FPVUS (American) and HP1 (European)) and attenuated, tissue culture adapted (FP9 (European)) FWPV strains. Instead of the predicted changes in immunomodulators as the mechanism of attenuation, members of multigene families, especially those encoding ankyrin repeat proteins were seen to be the drivers of attenuation [10]. Ankyrin repeat proteins are thought to be involved in poxvirus host-range [15], and have previously been implicated in the attenuation of sheeppox virus [16]. Over 49% (137 genes) of the CNPV genome and 38% (89 genes) of the FWPV genome are comprised of genes belonging to gene families, 51 and 31 of which are ORFs containing ankyrin repeats respectively [9,11]. These and other differences in immunomodulatory gene families encoded by avipoxviruses may account for the extensive variability in virulence, host range and host interaction [11].

FPVUS and CNPV are significantly divergent with amino acid identity between ORF homologues (55-74%) being similar to that observed between different ChPV genera [11]. The CNPV genome contains 39 ORFs not present in FWPV, 29 of which encode unique, hypothetical proteins. CNPV contains ORFs coding for two additional proteins involved in nucleotide metabolism (thymidylate kinase and the small subunit of ribonucleotide reductase), TNFR (cnpv086), an IL-10 like protein (cnpv018), cellular ubiquitin (cnpv096), a protein tyrosine phosphatase (cnpv085), a thioredoxin binding protein (cnpv149), and two Rep like proteins (cnpv153 and cnpv200). FWPV contains 15 ORFs not present in CNPV, 13 of which encode hypothetical proteins. Homologues of fpv217 and fpv250 are notably absent from CNPV, and are similar to ORFs from insect baculoviruses, and from avian herpes- and adenoviruses respectively.

Members of the *Columbidae* family, consisting of pigeons and doves are susceptible to avipoxvirus infection and cases have been reported in several Columbiform species including the mourning dove [17], laughing dove [18], white-tailed laurel-pigeon [19], rock pigeon [3,5], feral pigeon [5] and others [1]. Due to the host species based approach to avipoxvirus taxonomy, poxviruses infecting the Columbiformes are designated as Pigeonpox viruses (PGPV).

This study focuses on two avipoxviruses, one isolated from a Feral Pigeon (*Columba livia*) from Port Elizabeth, South Africa (FeP2) in 2011 and the other from an African penguin (*Spheniscus demersus*) housed at SANCCOB, a seabird rehabilitation centre, following an oil spill off the coast of Cape Town, South Africa (PEPV) in 1988 [8,20]. These locations are 790 km apart.

Isolation and preliminary characterisation of PEPV has been previously described [8,20]. Poxvirus infections have not been observed in wild African penguins living in coastal waters around Cape Town (Personal communication with Nola Parsons and Tertius Gous, SANCCOB). Mosquito netting was introduced at SANCCOB in 2008 to protect housed penguins from contracting avian malaria and there has been no evidence of pox infections since then, suggesting that the initial infections were transmitted by biting insects. It is worth noting that many pigeons nest in the SANCCOB enclosure and could well have been the primary host for this virus, which could have been transmitted to penguins via biting insects. It is therefore possible that the African penguin is not the primary host of PEPV.

When grown on the chorioallantoic membranes of embryonated 10–11 day old chicken eggs, the pigeonpox virus FeP2 caused greater cell proliferation and immune cell infiltration as compared to PEPV, CNPV, FWPV and some of the other avipoxvirus isolates investigated in our laboratory [5]. Furthermore, based on phylogenetic analysis of four conserved genetic regions, FeP2 groups in clade A, subclade A3 and PEPV belongs to clade A, sub-clade A2. FPVUS and FP9 group in clade A1 and CNPV groups in clade B1 [5]. Poxviruses isolated from the African, Magellanic and Humboldt penguins group in Clades A2, A3 (FWPV-like) and B1 (CNPV-like) respectively [6,8]. Columbiformes have been shown to be susceptible to viruses from clade A (Fowlpox-like) [3,5,21,22] and clade B (Canarypox-like) [3,4,23].

In this study we have sequenced the genomes of two novel South African avipoxviruses using the 454 (Roche, Life Sciences) and Ion Torrent (Life Technology) platforms. We present a purpose-designed bioinformatics pipeline for analysis of next generation sequence data of avian poxviruses and compare the different avipoxviruses sequenced to date with particular attention to the viruses isolated from a Feral Pigeon (Port Elizabeth, SA) and African Penguin (Cape Town, SA).

## Results

The FeP2 and PEPV genomes were assembled into contiguous sequences of 282,356 bp and 306,862 bp respectively. Because the terminal hairpin loops were not sequenced, for each genome the left most nucleotide was arbitrarily nominated base 1.

### Phylogenetic analysis of FeP2 and PEPV

**Figure 1:**
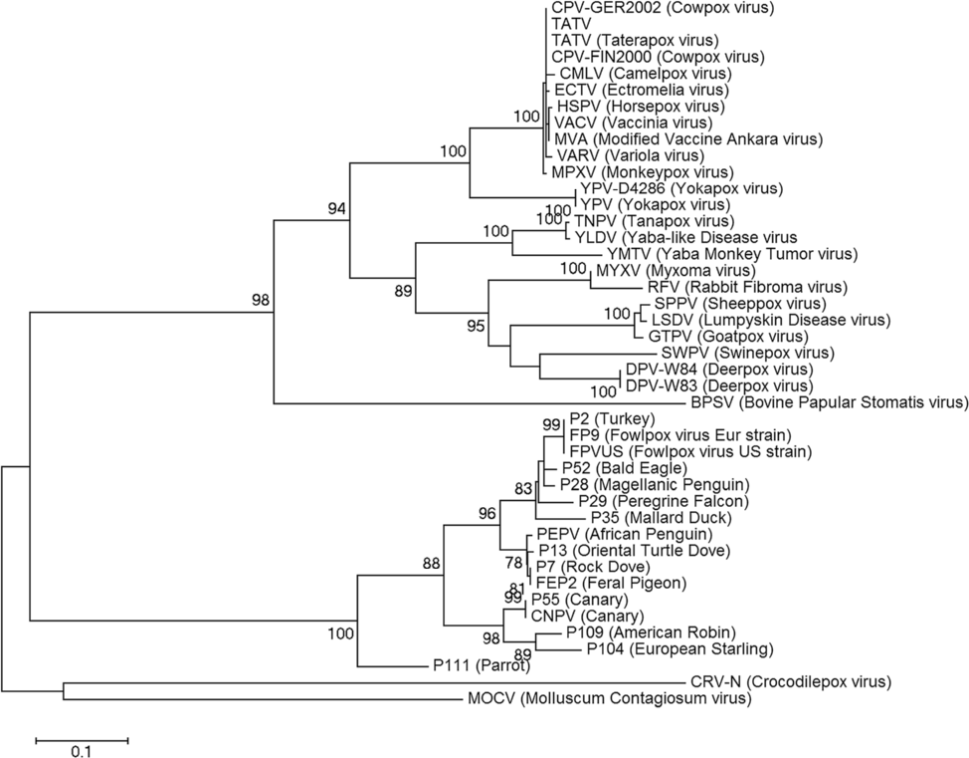
**Maximum likelihood phylogenetic trees based on the amino acid sequence alignment of the DNA polymerase genes from various poxviruses.** Multiple sequence alignment was performed with MUSCLE and the appropriate phylogenetic model was selected using ProtTest 2.4. The maximum likelihood phylogenetic tree based on the JTT matrix-based model (121) with 100 bootstrap replicates were performed in MEGA 5.10. Discrete Gamma distribution was used to model evolutionary rate differences among sites. The tree is drawn to scale, with branch lengths measured in the number of substitutions per site. All positions containing gaps and missing data were eliminated.

Phylogenetic analysis was performed using the DNA polymerase gene, a concatenated amino acid sequence of 17 translated ORFs and the entire genomic sequence (see Methods). All three analyses produced comparable tree topologies and the tree based on DNA polymerase is given in Figure 1. FeP2 and PEPV are most closely related to one another, grouping on the FWPV branch (Clade A), and separate from CNPV (Clade B).

### Comparisons of FeP2, PEPV, FWPV and CNPV DNA

**Figure 2:**
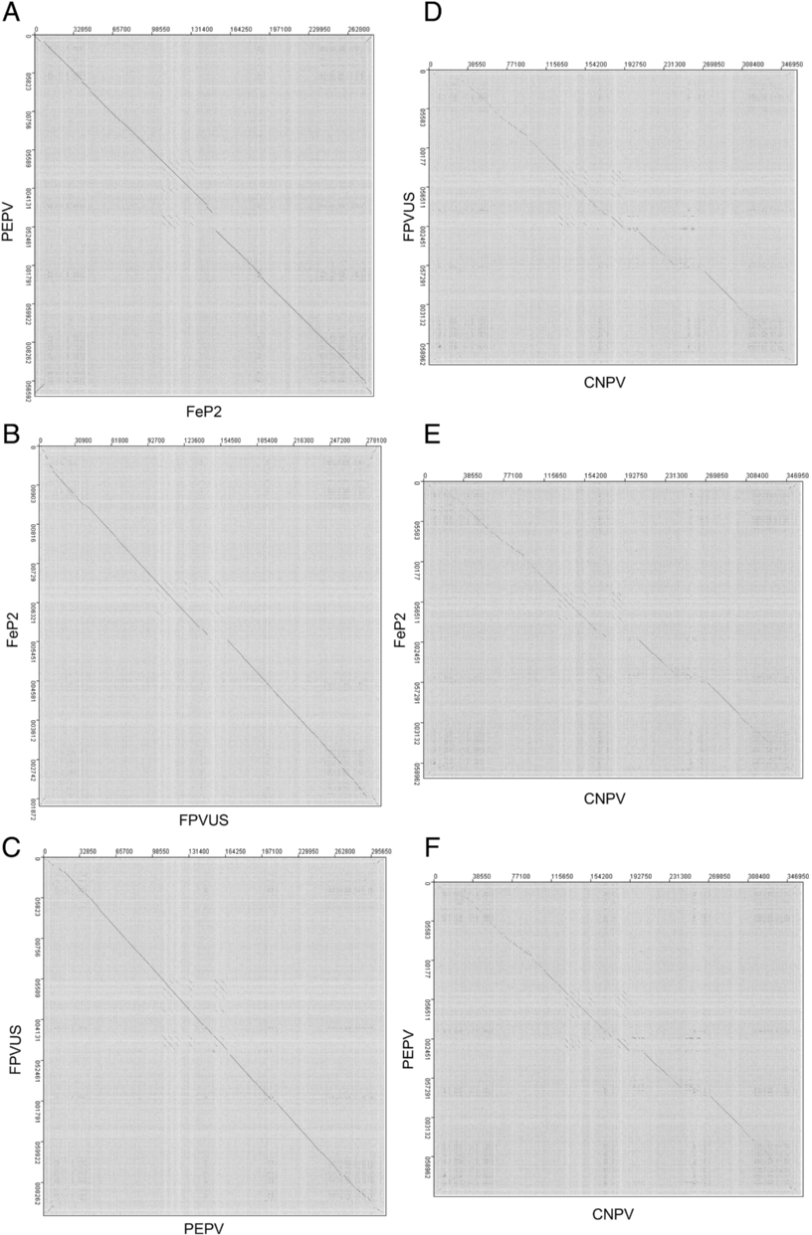
**Dotplot comparisons of Pigeonpox virus (FeP2), Penguinpox virus (PEPV), Fowlpox virus (FPVUS) and Canarypox virus (CNPV). A)** Dotplot comparing FeP2 (horizontal) and PEPV (vertical). **B)** Dotplot comparing FPVUS (horizontal) and FeP2 (vertical). **C)** Dotplot comparing PEPV (horizontal) and FPVUS (vertical). **D)** Dotplot comparing CNPV (horizontal) and FPVUS (vertical). **E)** Dotplot comparing CNPV (horizontal) and FeP2 (vertical). **F)** Dotplot comparing CNPV (horizontal) and PEPV (vertical). Dotplots were constructed using Jdotter (Brodie et al., 2004) [73]. Major deletions can be seen as breaks in the diagonal line. Lines perpendicular to the main diagonal in the top-right and bottom-left corners indicate the inverted terminal repeats (ITRs). The short diagonal lines, parallel to the main diagonal and near the centre of the plots, represent the members of the Variola virus B22R family.

The four genomic sequences of FeP2, PEPV, FWPV and CNPV were compared for nucleotide percentage identity. PEPV and FeP2 are more closely related to one another (94.3%) than they are to either FWPV (85.3% and 84.0% respectively) or CNPV (62.0% and 63.4% respectively). Dot plot analysis reveals a large deletion of 16 kbp in FeP2 compared to both of PEPV and FWPV (Figure 2A and B). Like the other avipoxviruses, FeP2 and PEPV have A/T rich genomes, both with 70.5% A + T content.

### Inverted Terminal Repeats (ITRs)

The FeP2 and PEPV genomes, like most other poxviruses, contain two identical inverted repeated sequences at their termini (ITR). These are 4,682 bp in FeP2 and 5,766 bp in PEPV, both of which are shorter than the ITRs of the other sequenced avipoxviruses (FPVUS: 9,520 bp, FP9: 10,158 bp and CNPV: 6,491 bp). Like FPVUS and CNPV, their A + T content within the ITRs is lower than average - 64.8% for FeP2 and 65.7% for PEPV. At the terminal ends of each ITR in FeP2 exists a 257 bp repeat region with 3.1 and 3.5 copies respectively of a 34 bp and 55 bp tandem repeat. In contrast, PEPV has a 165 bp repeat region containing 3.5 copies of a 47 bp tandem repeat. FPVUS contains a 1.7 kbp region with 42 copies of a 31-32 bp tandem repeat and CNPV contains at least 31, 9, and 7 copies of 17-, 41-, and 48-bp tandem repeats respectively within the terminal regions of each ITR [11]. The repeated sequences are unique for the different avipoxviruses.

### Overall arrangement of ORFs in genome

**Figure 3:**
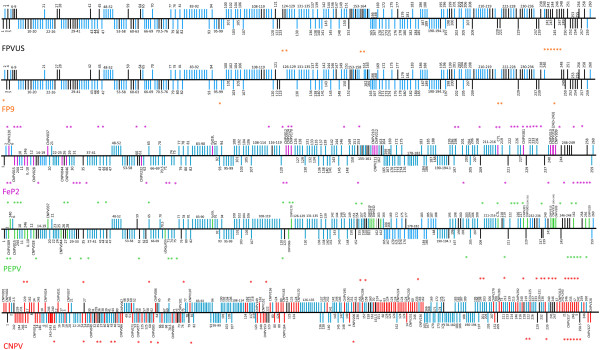
**Gene content of the 5 sequenced avipoxvirus genomes.** Fowlpox virus United States strain (FPVUS) is used as the reference genome against which other avipoxviruses (fowlpox virus FP9 strain (FP9), pigeonpox virus (FeP2), penguinpox virus (PEPV), canarypox virus (CNPV)) are compared. For each genome, the genes (vertical bars) are upwards when transcribed towards the right and downwards when transcribed towards the left. The genes that are shared amongst all 5 sequences are represented in blue on each genome. Genes shared amongst at least two viruses are represented as black. Differences, as compared to FPVUS, are represented in each genome as the same colour as the virus name. Deletions relative to FPVUS are indicated by an asterisk.

**Figure 4:**
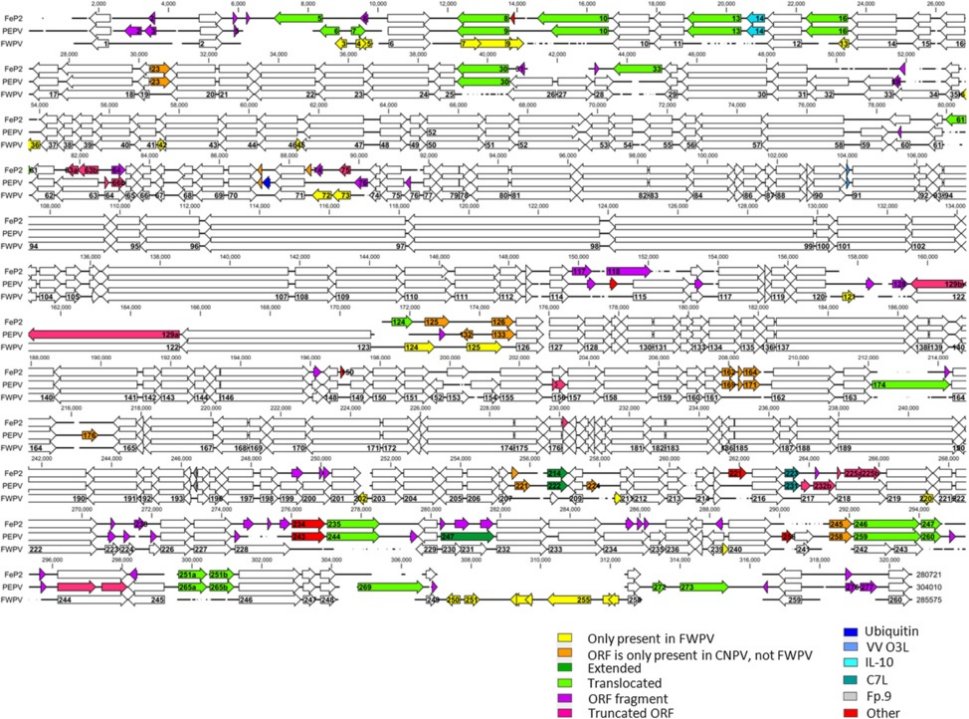
**A multiple sequence alignment (MUSCLE) and comparative ORF map of FeP2, PEPV and FPVUS genomes.** Blocks represent ORFs, with arrows depicting the direction of transcription. White blocks depict ORF homologues present in all three genomes at the same/similar location and differences between genomes are labelled and shown as coloured blocks in FeP2 and PEPV relative to FPVUS.

The genomes of FeP2 and PEPV have been compared to FPVUS, FP9 and CNPV in Figure 3. Figure 4 provides a multiple sequence alignment and comparative ORF map of FeP2, PEPV and FPVUS genomes. Clearly, there is conservation amongst the 5 sequenced avipoxviruses, but, at the same time, there are major differences between them. Unlike the orthopoxviruses, the avipoxviruses exhibit variation within the central core region where blocks of avipoxvirus genes have been inverted and/or transposed, suggesting that this genus is the most divergent of the ChPVs [9,11,24]. Comparison of gene orthologues indicate overall genomic synteny between FeP2, PEPV and FPVUS and genomic organisation is similar to that of other sequenced ChPVs [9,25,26]. Additional file 1: Table S1 lists all Open Reading Frames (ORFs) potentially coding for proteins of >30 amino acids for FeP2 and PEPV, and Figures 3 and 4 represent these graphically.

FeP2 contains 271 ORFs which have been annotated as putative genes and as orthologues of FPVUS and other genes (Additional file 1: Table S1, Figures 3, 4). These ORFs represent an approximate coding density of 82.5% and encode proteins between 34 and 1,937 amino acids (Additional file 1: Table S1). As published by Hendrickson et al., [27], an ORF was annotated as a whole gene if intact at its 5’ end with at least 80% the length of its orthologue. An ORF intact at its 5’ end but less than 80% of its orthologue length has been annotated as a truncated gene. Any ORF that has been disrupted at its 5’ end has been annotated as a fragmented gene, which is not expected to be transcribed and/or translated into a functional gene product [27]. Of these 271 ORFs, 11 have been annotated as truncated genes (Additional file 1: Table S1). A further 36 of these ORFs are fragmented forms of 28 larger orthologous genes (Additional file 1: Table S1). Therefore a total of 224 ORFs have been annotated as full length putative genes.

PEPV contains 284 ORFs which have been annotated here as putative genes (Additional file 1: Table S1) representing an approximate coding density of 85.4% and encoding proteins ranging between 34 and 1,922 amino acids long (Additional file 1: Table S1). A total of 242 of these ORFs have been annotated as whole genes, 14 as truncated genes (Table 1) and 28 as fragments of larger genes (Table 1).

**Table 1:** Deletions and disruptions in Pigeonpox virus (FeP2) and Penguinpox virus (PEPV) relative to Fowlpox virus United States Strain (FPVUS)

**FeP2**	**PEPV**	**Function**
**Deleted genes**		
fpv003	fpv003	C-type lectin family
fpv004	fpv004	hypothetical protein
fpv005	fpv005	Efc family
fpv007	fpv007	hypothetical protein
fpv008	fpv008	C-type lectin family
fpv009	fpv009	hypothetical protein
fpv013	fpv013	hypothetical protein
fpv027	I	G-protein coupled receptor family
fpv032	I	Dnase II
fpv033	I	α-SNAP
fpv036	fpv036	hypothetical protein
fpv042	fpv042	hypothetical protein
fpv045	fpv045	hypothetical protein
F	fpv072	β-NGF
T	fpv073	IL-18 binding protein
fpv076	F	β-NGF
fpv115	I	Ankyrin repeat family
fpv121	fpv121	CC-chemokine family
fpv122	T	B22R
fpv123	I	B22R
fpv124	fpv124	N1R/p28 family
fpv125	fpv125	V-type Ig Domain
fpv152	I	HT-motif
I	fpv153	hypothetical protein
fpv154	I	hypothetical protein
fpv210	fpv210	hypothetical protein
fpv220	fpv220	hypothetical protein
fpv224	F	Ankyrin repeat family
fpv225	F	hypothetical protein
fpv238	fpv238	hypothetical protein
fpv250	fpv250	US ORF2
fpv251	fpv251	Serpin
fpv252	fpv252	hypothetical protein
fpv253	fpv253	C-type lectin family
fpv254	fpv254	hypothetical protein
fpv255	fpv255	C4L/C10L-like family
fpv256	trans	Efc family
fpv257	fpv257	hypothetical protein
**Disrupted genes**		
fpv026 fragmented	I	ankyrin repeat
fpv028 truncated	I	hypothetical protein
fpv034 fragmented	fpv034 fragmented	ankyrin repeat
I	fpv060 fragmented	cc-chemokine
fpv063 truncated	I	hypothetical protein
fpv064 fragmented	fpv064 truncated	glutathione peroxidase
fpv072 fragmented	-	β-NGF
fpv073 truncated	-	IL-18 binding protein
-	fpv076 fragmented	β-NGF
-	fpv122 truncated	B22R
I	fpv156 truncated	hypothetical protein
fpv162.2 fragmented	I	ankyrin repeat
fpv177 truncated	I	hypothetical protein
fpv199 fragmented	I	V-Type Ig Domain
fpv200 fragmented	I	V-Type Ig Domain
fpv217 fragmented	fpv217 truncated	hypothetical protein
fpv218 truncated	I	ankyrin repeat
fpv223 fragmented	fpv223 fragmented	ankyrin repeat
-	fpv224 fragmented	ankyrin repeat
-	fpv225 fragmented	ankyrin repeat
fpv228 fragmented	fpv228 fragmented	ankyrin repeat
fpv234 fragmented	I	ankyrin repeat
fpv239 fragmented	I	C-type lectin
I	fpv244 truncated	ankyrin repeat
fpv245 fragmented	I	ankyrin repeat
I	fpv249 fragmented	hypothetical protein

Where a gene is deleted or disrupted in only one virus (FeP2/PEPV), then the status of the other is given as either Intact (I), Truncated (T), Fragmented (F) or Translocated (Trans). Putative functions for the deleted genes have been given where possible.

Relative to the FPVUS genome, FeP2 and PEPV have 36 and 25 deleted ORFs respectively (Table 1). FeP2 contains 34 inserted ORFs relative to FPVUS, comprising 12 fragments of larger genes, 2 truncated genes and 20 intact genes (Table 2). Similarly, PEPV contains 42 inserted ORFs, which include 17 gene fragments, 2 truncated genes and 23 intact genes (Table 2). A total of 214 intact ORFs are shared between PEPV and FeP2 throughout the genome and 5 of these do not have any functional orthologue in FPVUS (Additional file 1: Table S1). Amongst PEPV, FeP2 and FWPV, the most conserved ORF is the orthologue of fpv103 (fep105, pep107 and VACV F17R) which encodes the DNA binding virion core phosphoprotein and shares 100% amino acid identity amongst the three viruses.

**Table 2:** Insertions in Pigeonpox virus (FeP2) and Penguinpox virus (PEPV) relative to Fowlpox virus United States Strain (FPVUS)

**FeP2**	**PEPV**	**Function**
Fragmented cnpv021	-	ankyrin repeat
-	Fragmented cnpv319	ankyrin repeat
-	Fragmented cnpv310	ankyrin repeat
Fragmented cnpv006	Fragmented cnpv006	hypothetical protein
cnpv320	-	Ig Domain protein
Fragmented cnpv309	cnpv309	ankyrin repeat
-	fpv250	hypothetical
cnpv015	cnpv015	ankyrin repeat
Fragmented Trichomonas vaginalis	-	ankyrin repeat
fpv244	fpv244	ankyrin repeat
fpv246	fpv246	ankyrin repeat
IL-10 (Ficedula albicollis)	IL-10 (Ficedula albicollis)	IL-10
cnpv028	cnpv028	ankyrin repeat
cnpv037	cnpv037	hypothetical protein
cnpv044	cnpv044	ankyrin repeat
cnpv046	-	ankyrin repeat
cnpv232	-	CC-chemokine-like protein
cnpv095	cnpv095	hypothetical protein
-	Ubiquitin	Ubiquitin
Fragmented cnpv098	-	hypothetical protein
-	fragment cnpv012	hypothetical protein
fp03L	fp03L	vaccinia 03 L ortholog
Fragmented cnpv011	-	ankyrin repeat
Fragmented cnpv004	Fragmented cnpv004	ankyrin repeat
-	Fragment Pfs, Nacht protein Neosartorya Fischeri	ankyrin repeat
-	truncated cnpv221	N1R/p28-like protein
-	fragmented cnpv165	N1R/p28-like protein
-	fragmented cnpv162	TGF-β
-	cnpv086	TNFR-like protein
cnpv012	-	hypothetical protein
cnpv224	-	hypothetical protein
cnpv170	cnpv170	thymidilate kinase
Fragmented E. Bacterium	-	hypothetical
cnpv210	cnpv210	N1R/p28-like protein
cnpv211	cnpv211	hypothetical protein
cnpv212	cnpv212	N1R/p28-like protein
-	Fragmented cnpv041	ankyrin repeat
Truncated cnpv279	Fragmented cnpv279	beta-NGF protein
-	fragmented cnpv283	CC-chemokine-like protein
Fragmented protein (X. tropicalis)	-	hypothetical
Tanapox 67R	Tanapox 67R	Host Range (C7L-like)
Fragmented O.tsutsugamushi str	Fragmented O.tsutsugamushi str	ankyrin repeat
cnpv301	cnpv301	ankyrin repeat
-	Fragmented GTPV gp138	hypothetical
cnpv313	cnpv313	Ig Domain protein
-	cnpv014	Ig Domain protein
fragment cnpv320	fragment cnpv320	Ig Domain protein
truncated cnpv014	truncated cnpv014	Ig Domain protein
-	fpv162	ankyrin repeat
-	cnpv320	Ig Domain protein
-	fragment cnpv006	hypothetical protein
cnpv021 Frag	-	ankyrin repeat
-	cnpv310 Frag	ankyrin repeat
-	cnpv319 Frag	ankyrin repeat

Inserted genes have been listed if they are present in FeP2 or PEPV in an unexpected site as predicted by genomic synteny with the FPVUS. Where no homology to FPVUS genes is present, the name of the best BlastP hit has been used.

Both PEPV and FeP2 lack sequences similar to the reticuloendotheliosis virus (REV) observed in FPVUS and other avipoxvirus strains [9,28,29].Although the FeP2 and PEPV genomes are closely related and syntenic to the FWPV genome overall (Figure 2), when compared to each other, and to FWPV, these viruses display differences in gene content throughout their genomes. Of particular note is the large 16 kbp deletion in FeP2 relative to both PEPV (Figure 2A) and FPVUS (Figure 2B).

### Conserved genes

There are 179 genes that are conserved amongst all five sequenced avipoxviruses, which have been highlighted in blue in Figure 3. These include genes that lie within the more variable terminal regions of the genome. These avipoxvirus core genes were defined as such if intact orthologues of the genes were present at least once in all five avipoxvirus sequences. The 179 APV core genes include the 90 core genes conserved in all ChPVs which are involved in essential functions such as replication, transcription and virion assembly [24,30] (indicated in bold and italic in Additional file 1: Table S1). There are an additional 89 genes conserved in all avipoxviruses with a variety of different functions (indicated in bold in Additional file 1: Table S1).

### Gene families

Avipoxviruses contain extensive gene families, which vary greatly between different species. In CNPV, these comprise over 49% of the genome, whereas they encompass 38% of the FWPV genome [9,11]. In CNPV and FWPV, these gene families account for much of the variation between the two genomes [11]. Intact members of gene families comprise 33% of the FeP2 and PEPV genomes. Table 3 summarises the differences in the number of intact gene family proteins found in the five sequenced avipoxvirus genomes.

**Table 3:** A summary of the numbers of intact gene family proteins found in different poxviruses

	**FeP2**	**PEPV**	**FPVUS**	**FP9**	**CNPV**	**VACV**
Ankyrin repeat proteins	26	33	31	22	51	17
B22R	4	5	6	5	6	1
N1R/p28	11	11	10	8	26	0
C4L/C10L	2	2	3	3	3	3
CC chemokine	4	1	4	4	5	2
C-type Lectin	4	7	9	6	11	2
G protein-coupled receptor gene family	2	3	3	2	4	0
HT Motif	4	5	6	6	5	0
Ig-like domain protein	4	6	5	4	9	3
Serpin	4	4	5	5	5	2
Efc family	1	1	3	2	2	0
TGF-β	1	1	1	1	5	0
β-NGF	0	0	2	2	2	0
interleukin 18 (IL-18)-binding protein	0	1	1	1	3	1

A summary of the numbers of intact gene family proteins in pigeonpox virus (FeP2), penguinpox virus (PEPV), fowlpox virus US strain (FPVUS), fowlpox virus FP9 strain (FP9), canarypox virus (CNPV) and vaccinia virus (VACV). “0” is given where no member of the gene family is present in the respective virus.

### Gene translocations and duplications

Analysis of the FeP2 and PEPV genomes revealed several occurances of gene duplication and translocation relative to FWPV. In some instances the postion of the gene was closer to that observed in CNPV than FWPV. A second copy of the Ankyrin repeat family gene, fpv244 orthologue (fep010; pepv010; cnpv009) is found in the equivalent place in the left hand region of each African avipoxvirus genome.

### Disrupted and deleted genes

Relative to FPVUS, FeP2 contains 5 truncated FPVUS genes and the fragmented forms of 13 FPVUS orthologues. PEPV contains 5 truncated genes and the ORF fragments of 8 FPVUS orthologues (Additional file 1: Table S1). Both FeP2 and PEPV contain truncated or fragmented remains of ORFs with similarity to CNPV (Table 2).

Relative to the FPVUS genome, FeP2 and PEPV have 36 and 25 deleted ORFs respectively (Table 1) that are absent from any potential coding regions of their genomes.

### Inserted genes

**Figure 5:**
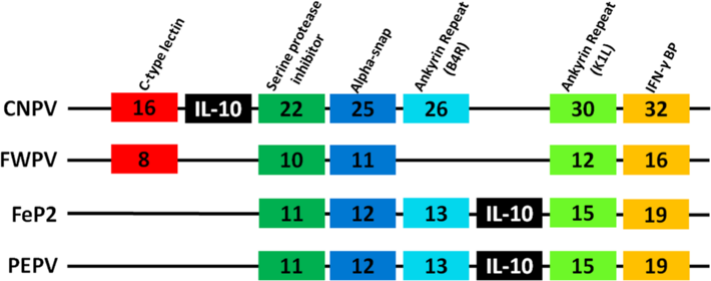
**Analysis of Interleukin-10 (IL-10) in Avipoxviruses.** Gene synteny analysis depicting syntenic ORFs (that are present in at least 2 of the depicted genomes) highlighting the location of IL-10.

Relative to FWPV, the PEPV and FeP2 genomes both contain several inserted genes (Table 2). PEPV, but not FeP2 or FWPV [9], has orthologues of cnpv086 (pepv132) which encodes a protein similar to **Tumour necrosis factor receptor** (TNFR) [11] and a 77 amino acid long orthologue of **Ubiquitin** (pepv074) which is most similar to human ubiquitin, S5a ubiquitin-interacting motif-1 (UIM-1) (Accession: 1YX5_B) [31]. Both PEPV (pepv133) and FeP2 (fep126) contain orthologues of **thymidylate kinase** (TMPK) (cnpv170; VACV A48R) (Table 2). FeP2 and PEPV both encode a putative **Interleukin-10** (IL-10) gene with 80.9% amino acid identity between them (fep014, pepv014) and limited similarity to the CNPV orthologue (22.1% and 23.3% aa identity respectively) (not shown). Amino acid identity between all available avian IL-10 genes and PEPV and FeP2 fall between 32.5% and 27.1%, however for CNPV the identities are significantly lower (23.1%-19.0%). The PEPV, FeP2 and CNPV IL-10 genes are most similar to that of a Collared Flycatcher (*Ficedula albicollis*) sharing 32.5%, 30.2% and 23.1% amino acid identity respectively (not shown). FWPV does not encode an IL-10 gene [9]. The respective positions of FeP2, PEPV and CNPV IL-10 orthologues were analysed to determine the possibility of these genes existing in the three genomes as a result of independent horizontal gene transfer events (Figure 5). Based on this analysis, it is possible that there were two independent transfer events into avian poxviruses, one into CNPV and another into the progenitor of PEPV and FeP2.

PEPV and FeP2 both encode homologues of Tanapox virus (TANV) and Yaba-Like Disease virus (YLDV) 67R which are orthologues of **VACV C7L**. These C7L-like genes (pepv231 and fep223) are in the equivalent genome positions, between orthologues of fpv216 (pepv230; fep222) and fpv217 (pepv232 [truncated]; fep224 [fragmented]) (Additional file 1: Table S1, Figures 3 and 4).

### Comparison of FeP2 and PEPV to attenuation and lineage specific mutations

In order to determine lineage specific mutations, Laidlaw and Skinner [10] determined the sequence of HP1 (European strain), the virulent parent of FP9, at all loci where FP9 differed from FPVUS (American strain) [10]. Here we compared nucleotide changes in PEPV and FeP2 to 44 genomic positions influencing amino acid composition of 25 open reading frames between European and American strains as previously identified [10]. In 15 and 17 of the places, FeP2 and PEPV were the same as HP1 (European lineage) respectively. In 18 and 17 of the places, FeP2 and PEPV were the same as FPVUS (American lineage) respectively. PEPV and FeP2 differ from both HP1 and FPVUS in 9 and 10 places respectively.

## Discussion

Genomic sequences have been determined for two novel South African avipoxviruses. The DNA sequences of PEPV and FeP2 are significantly different from those of CNPV (approx. 63% identity) and FWPV (approx. 85% identity). In Orthopoxviruses (OPVs), the internal region of different species share at least 96% identity when compared at the nucleotide level, while different strains from the same species share at least 99% nucleotide sequence identity [27-33]. These identities have been listed as criteria for establishing poxvirus taxonomy [33]. Therefore, according to the identities between PEPV, FeP2 and FPVUS genomes (94.4% between PEPV and FeP2, 85.3% between PEPV and FPVUS and 84.0% between FeP2 and FPVUS), as well as the differences in phylogenetics and growth characteristics between these two viruses [5], one can deduce that PEPV and FeP2 are separate species of APV.

Laidlaw and Skinner [10] identified geographical lineage-specific mutations which distinguished European fowlpox viruses, FP9 and HP1 from the American strain FPVUS. A comparison of PEPV and FeP2 with the published FWPV sequences at these sites shows that PEPV and FeP2 share lineage-specific mutations with both HP1 and FPVUS. The South African viruses are no more closely related to either the American or the European virus. These results suggest that FeP2 and PEPV originate from a common ancestor that diverged relatively recently from a FWPV-like progenitor which was more distantly related to the CNPV-like progenitor. It is important to note that the divergence between avipoxvirus species is comparable to that between some poxvirus genera and the different species within the OPV genus are highly similar.

FeP2 and PEPV are most closely related to FWPV. Despite this, relative to FWPV, the PEPV and FeP2 genomes both contain several genes that are more closely related to CNPV throughout their genomes. FeP2 and PEPV encode 16 and 15 intact CNPV orthologues respectively (Table 3), five of which are not in FWPV, including a TNFR-like protein (cnpv086, pepv132) and a TMPK protein (cnpv170, fep126, pepv133) (Additional file 1: Table S1 and Table 3). The remainder are more similar to CNPV orthologues but are present in FWPV. Rather than being acquired through horizontal gene transfer or recombination, these genes are most probably from a common ancestor. Their existence suggests that FeP2 and PEPV viruses diverged prior to the present day FWPV, somewhere between the CNPV and FWPV branches. Several of these CNPV-like genes exist as gene fragments (Table 2), which is indicative of their gradual loss through progressive mutation, rearrangements and/or deletions over many rounds of virus replication.

There are several instances of gene duplication and translocation in FeP2 and PEPV relative to FWPV (Figures 3 and 4). Most of these involve genes containing ankyrin repeats. Virulent FWPV contains 31 ankyrin repeat containing genes and attenuated FP9 contains 22 of these genes. PEPV contains 33 ankyrin repeat-containing genes whereas FeP2 contains only 26 (Additional file 1: Table S1; Table 3). It is postulated that loss of these genes may be responsible for the attenuation of the virus [9,10]. Thus the reduced numbers of intact ankyrin repeat genes observed in FeP2 compared to PEPV would suggest that this virus is less virulent. On the other hand, the expansion of the number of ankyrin repeat genes in certain avipoxvirus strains could represent the forming of adaptive genomic accordions, similar to that of the K3L gene in OPV, the formation of which has been described to play an adaptive role in these viruses [34].

The most striking difference between FeP2 and the FWPV-like avipoxviruses, is a large deletion of ~16 kbp from the central, usually conserved, region of the genome. ORFs corresponding to fpv121 (cc-chemokine family), fpv122 and fpv123 (VAR B22R family), fpv124 (N1R/p28 family) and fpv125 (V-type Ig domain gene family) are deleted in FeP2 at this site. The existence of gene fragments and insertions on the borders of this deletion, as well as in the equivalent regions in FWPV and PEPV, suggests that this is a “hotspot” of genetic change, the mechanism of which is uncertain.

Relative to the FPVUS genome, FeP2 and PEPV have 36 and 25 deleted ORFs respectively (Table 1). Most of the genes deleted in PEPV and FeP2 are members of multi-gene families, the disruption of which has been implicated in the attenuation of poxviruses [10], however there are several other deletions of significant ORFs. Of interest, the orthologue of fpv032 encoding a DNase II has been deleted in FeP2 but is in intact in PEPV. In FWPV, fpv032 represents the large subunit of cellular DNase II [9] and cellular DNase II is thought to function in DNA catabolism during apoptosis [35]. Additionally, the orthologue of fpv033 encoding one of the two α-SNAP genes present in FWPV, is absent from FeP2 but intact in PEPV (pepv038). Eukaryotic α-type soluble NSF attachment proteins (α-SNAP) are involved in vesicular transport through the Golgi apparatus and for exocytosis [36]. The fpv033 gene has been shown to be conserved in FWPV strains but non-essential to viral replication and it is thought to be involved in virus-host interactions [37]. Although the fpv033 gene orthologue has been deleted in FeP2, a second α-SNAP-like gene (fpv011, pepv012) exists in the genome which exhibits 34.0% and 33.8% amino acid identity with fpv033 and pepv038 respectively.

FWPV contains two genes with homology to cellular β-nerve growth factor (β-NGF) (fpv072 and fpv076). In FeP2, the orthologue of fpv076 is completely deleted and the orthologue of fpv072 (fep074) is fragmented (Table 1). Conversely, in PEPV, the orthologue of fpv072 is completely deleted and the orthologue of fpv076 (pep079) is fragmented (Table 1). An additional β-NGF-like gene is observed in a truncated form in FeP2 (fep213) and as a fragmented gene in PEPV (pepv221) (Table 2). These are most similar to the CNPV gene cnpv279, which exists as a fragment in FWPV [11]. The FeP2 ORF, fep213 is truncated to 67 amino acids by the introduction of a premature stop codon, where the intact cnpv279 orthologue is 169 amino acids. It is unknown whether this protein would be functional in FeP2 but it is unlikely that PEPV encodes a functional β-NGF protein. β-NGF has proinflammatory properties and is produced by cells in response to infection, injury and stress [38]. FPV encoded β-NGF is thought to be involved in promoting survival in infected cells and could have a role in inhibiting antiviral immune responses in the host [9]. Previously, it was speculated that the absence of a β-NGF gene in an avipoxvirus genome could be partly responsible for the decreased inflammatory response observed in PEPV-infected CAMs as compared to FeP2 [5].

An orthologue of fpv073 encoding an IL-18 binding protein has been deleted in PEPV. In FeP2 this ORF (fep075) has been truncated due to a 1 bp deletion resulting in a premature stop codon. IL-18 is a multifunctional pro-inflammatory cytokine that induces interferon gamma (IFN-γ) production, Th-1 responses and NK cell activity [39]. It is modulated in vivo by IL-18 binding protein (IL18BP) via a negative feedback mechanism [40]. IL18BP homologues are encoded by many poxviruses [9,11,41,42] and have been found to inhibit IL-18-dependent IFN-γ production [43]. In FWPV, fpv073 is thought to have an anti-inflammatory function [9].

PEPV contains an orthologue of cnpv086 (pepv132) which encodes a protein similar to TNF receptor [11]. This ORF is absent from FeP2 (Additional file 1: Table S1) and FWPV [9]. Tumour Necrosis Factor (TNF) is a pro-inflammatory cytokine which is involved in induction of cytokines, cell proliferation, differentiation, necrosis and apoptosis [44]. TNF -induced cellular responses are mediated by one of two types of TNF receptors [44]. Several poxviruses encode TNFR homologues which function to modulate TNF-induced anti-viral responses [11,45-47].

PEPV (pepv133) and FeP2 (fep126) both contain an orthologue of thymidylate kinase (TMPK) (cnpv170; VACV A48R), which is absent in FWPV [9]. This is the only difference found with respect to the complement of nucleotide metabolism genes. Interestingly, despite being within the conserved central region of the genome, the TMPK gene orthologues occur within a region of PEPV and FeP2 that is highly variable compared to FWPV. In addition, the CNPV orthologue insertions that occur within this particular variable region in FeP2 and PEPV occur in different sites across the CNPV genome. The higher than average amino acid identity shared between FeP2 and PEPV (90.8%) and the CNPV genomes (81.6% and 80.2% respectively), suggests that this TMPK gene is necessary and conserved in these viruses. CNPV, FeP2 and PEPV are the only poxvirus species outside of the OPV genus to contain a TMPK homologue. VACV encodes TMPK (A48R) that is 42% identical to human TMPK [48]. TMPK catalyzes an important step in the biosynthesis of (deoxy) thymidine triphosphate and is essential for cell metabolism [49]. In CNPV, FeP2 and PEPV, the presence of a TMPK gene suggests a different optimization of cellular nucleotide pools as compared to FWPV and probably affects cell and tissue tropism in the different viruses [11].

PEPV contains a 77 amino acid long orthologue of Ubiquitin (pepv074) which is most similar to human ubiquitin (97% BLASTp identity), S5a ubiquitin-interacting motif-1 (UIM-1)(Accession: 1YX5_B). CNPV also contains an intact ubiquitin orthologue (cnpv096) (86.1% amino acid identity to pepv074) [11]. FeP2 (nt81,771-81,622) and FPVUS (nt74,550-74,220) encode the fragmented remains of an ubiquitin gene but no open reading frame is observed in either genome [9]. In all four genomes these Ubiquitin ORFs or sequence remains are in the same location (between orthologues of fpv070.5 and fpv071). Ubiquitin is a highly conserved 76 amino acid protein with diverse cellular functions effected by means of marking proteins for intracellular signalling [50]. Ubiquitin proteins are thought to affect the diverse ubiquitination-mediated cellular functions, which may impact on CNPV and PEPV virulence and host range [11]. Other than CNPV and PEPV, ubiquitin genes have only been found in two insect poxviruses, Melanoplus sanguinipes (MSEV) [51] and Amsacta moorei (AMEV) [52]. Virus-encoded ubiquitin genes have also been identified in the *Baculoviridae*, including in the *Autographa californica* nuclear polyhedrosis virus (AcNPV) [53]. In AcNPV, disruption of the ubiquitin gene was shown to have no effect on virus viability but to cause a decrease in virion budding and total infectious particle production [53]. The ubiquitin encoding genes in PEPV and CNPV may have an effect on virus production and budding but this remains to be determined.

FeP2 and PEPV both encode a putative Interleukin-10 (IL-10) gene (Figures 3 and 4). Putative orthologues of IL-10 are also found in ORF virus, Bovine Papular Stomatitis Virus (BPSV) [54], Lumpy skin disease virus (LSDV) [55] and Yaba-Like disease virus (YLDV) [56]. IL-10 is a cytokine that has both immunostimulatory and immunosuppressive functions [57] and the ORF virus IL-10 orthologue has been shown to be immunomodulatory in function [58]. The IL-10 genes encoded by PEPV and FeP2 may also be involved immune evasion, however, because of the divergence from host IL-10 proteins (20-31% amino acid identities to various avian IL-10 genes), like cnpv018, these genes may have a novel biological function [11].

The IL-10 gene is likely to have been incorporated into poxvirus genomes via horizontal gene transfer (HGT) [59]. Bratke and McLysacht [59] made use of comparative genomic information and synteny conservation to investigate HGT in pox genomes [59]. Here they found that the IL-10 orthologue encoded by CNPV was in an unexpected location as compared to other poxviruses (yatapox and capripox) but because of the lack of conservation at this region, no conclusion could be drawn about the transfer event into CNPV. An analysis of the genome synteny of avipoxviruses in the region of the IL-10 gene shows that the CNPV IL-10 is in a different location despite being in a similar region to FeP2 and PEPV (Figure 5). Although this region is not well conserved between genera, it appears to be relatively well conserved between avian poxviruses making it possible that there were two independent transfer events into avian poxviruses, one into CNPV and one into the common ancestor of FeP2 and PEPV.

PEPV and FeP2 both encode a gene with homology to Tanapox virus (TANV) and Yaba-Like Disease virus (YLDV) 67R which are orthologues of VACV C7L and not found in FWPV or CNPV. The poxvirus C7L family of host range genes functions by mediating poxvirus host range and antagonising the host defence system [60]. VACV C7L orthologues are found in all orthopoxviruses and most mammalian poxviruses, with the exception of molluscum contagiosum virus and parapoxviruses [61]. This is the first report of a C7L-like gene in avipoxviruses. In mammalian cells, C7L has been shown to inhibit apoptosis [62], antagonise the anti-viral effects of type 1 interferons (IFNs) and Interferon Regulatory Factor 1 (IRF-1) [63] and can antagonize the dsRNA-activated protein kinase (PKR) pathway by inhibiting the phosphorylation of eIF2a [64]. It has previously been suggested that C7L orthologues are an important adaptation of mammalian poxviruses for replication in mammalian hosts [63]. The presence of C7L orthologues in PEPV and FeP2 confounds these previous observations but the function of this gene in these two avipoxviruses remains to be determined. The limited amino acid identity may suggest a novel function of these genes as compared to other C7L orthologues. However, YLDV 67 L shows only 28-30% identity to the VACV C7L protein but has been shown to function equivalently in supporting VACV replication in mammalian hosts [61].

## Conclusions

The genome sequences of FeP2 and PEPV have greatly added to the limited repository of genomic information available for the Avipoxvirus genus. In the comparison of FeP2 and PEPV to existing sequences, FWPV and CNPV, we have established insights into African avipoxvirus evolution. Although FeP2 and PEPV are more closely related to FWPV, the presence of whole or disrupted genes with similarity to CNPV genes that are absent in FWPV, suggests that FeP2 and PEPV originate from the common ancestor of CNPV and FWPV. The presence of an intact gene in CNPV, FeP2 and PEPV where the FWPV counterpart is fragmented into two ORFs further supports this theory as fragmented genes represent the gradual loss of genetic information during the process of virus evolution. Additional genome sequences of avipoxviruses would help to define avipoxvirus evolution as a whole.

The ongoing search for an ideal vaccine vector makes this work relevant. Future work could focus on how these two avipoxviruses differ from the well characterized FWPV and CNPV with respect to immune activation and foreign gene expression.

## Methods

### Viral DNA isolation

FeP2 and PEPV were grown on the chorioallantoic membranes (CAMS) of embryonated 10–11 day old chicken eggs and virus purified as described previously [5].

For 454 sequencing, genomic DNA was extracted using a method described previously [5] with an additional incubation with DNase (25 U/100 ul virus) at 55°C for 1 hr and subsequent inactivation of DNase at 80°C for 30 minutes, prior to treatment with proteinase K. Also, following the first phenol extraction, the virus preparation was treated with RNAse (100 μg/μl) and incubated at 37°C for 1 hour.

For Ion Torrent sequencing, special care was taken to prevent host (chicken) chromosomal and mitochondrial DNA contamination of the viral DNA preparation. Before DNA extraction virus preparations were treated with equal volumes of Vertrel^®^ (1,1,1,2,3,4,4,5,5,5-decafluoropentane) (DuPont) which is a Freon substitute that has been shown to separate virus from infected cellular debris in some viral purification methods [65]. The virus preparations were then freeze/thawed three times to lyse any remaining cells, and then treated with DNAse prior to lysis of the virions. DNA extraction was then performed as described previously [5].

### DNA sequencing and bioinformatics

High quality viral DNA was sequenced using a Roche 454 GS Junior system as per manufacturer’s instructions. Primary sequence analysis was performed using the GS Junior Software version 2.5p1 and de novo assembly was done using GS De Novo Assembler software and CLC Genomics Workbench. For Ion Torrent sequencing [66], DNA was sequenced using a 316 chip on an Ion Torrent Personal Genome Machine (PGM) (Life Technologies) at the Central Analytical Facilities, Stellenbosch University. The Covaris S2 system was used for physical shearing.

To offset possible remaining host DNA contamination, we made use of a customized bioinformatics pipeline as described here (Additional file 2). For 454 data, the SFF file was converted to FastQ in Galaxy, and filtered such that only reads shorter than 500 nt and with a mean QC > 20 were included. For the Ion torrent data, primary analysis was performed in Torrent Suite version 3.2.1. Firstly, the Raw SFF file was submitted to SFFTrim (Torrent Suite 3.2.1) and reads were trimmed when the average base quality values in a window size of 10 were less than 25 (Q-value of 25). Reads shorter than 50 nt were discarded. Reads were trimmed of adaptor sequences and filtered to remove polyclonal reads and trimmed to remove poor quality bases at the 3’ end of long reads.

These two datasets were then mapped to the Chicken (*Gallus gallus*) genome (WASHUC2 assembly) with Newbler 2.6 to filter out possible host contamination. Read IDs that did not map to the Chicken genome were obtained from the Newbler output and these unmapped reads were extracted from the original SFF files using Linux commandline tools and SFFFile that forms part of the 454 NGS data analysis software. The resulting SFF files were used as input data for de novo assembly. De novo assembly was performed in Newbler 2.6 using default parameters, as well as in Mira (Version 3.4.0) where de novo assembly was assisted by a reference genome (FPVUS).

For the FeP2 data, the Roche 454 sequencing resulted in 93,654 reads with an average length of 404 bp. Following filtering, only 2,369 (2.5%) of these reads did not map to the chicken genome and met the quality requirements. Ion Torrent sequencing resulted in a total of 3,239,283 reads with a mean read length of 191 bp. The filtered data set contained 1,068,645 reads or 32.9% of the total raw reads. Newbler assembly resulted in 7 contigs greater than 500 bp (278,380 bases total). MIRA assembly also resulted in 7 contigs greater than 500 bp (279,145 bases total). The assemblies were merged using the Genome Assembler, Reconciliation and Merging (GARM) version 0.70 meta assembler pipeline which resulted in 3 contigs and the remaining gaps were closed by PCR and Sanger sequencing and visual inspection. Raw PGM reads were then mapped back to the draft sequence. A total of 3,142,379 raw reads mapped to the draft sequence (97.0% of total) resulting in an average coverage of 2,129.9 X. Remaining gaps between contigs were closed by PCR and Sanger sequencing and raw reads were mapped back to the draft sequence using CLC Genomics workbench 4.7.2 and TMap as part of the Torrent Suite (Version 3.2.1) software.

For the PEPV data, Ion Torrent sequencing resulted in 3,197,371 reads with a mean read length of 203 bp. The filtered data set contained 1, 119,080 reads or 35.0% of the total raw reads. MIRA assembly resulted in one contig of 301,453 bp. PCR and Sanger sequencing were used to find and confirm the location of the inverted terminal repeats.

Open reading frames (ORFs) longer than 30 amino acids with a methionine start codon (ATG) and less than 50% overlap to other ORFs were called using the CLC Genomics Workbench (CLC) ORF analysis tool as well as with an Integrated Services for Genomic Analysis (ISGA) pipeline which makes use of GLIMMER3 [67], BLAST, HMMer2 [68] and other protein coding sequence and annotation software described by Hemmerich, et al. [69]. Similarity searches including nucleotide (BLASTn) and Protein (BLASTp) BLAST analyses were performed on every ORF and ORFs were annotated as potential genes if they shared significant sequence similarity to known viral or cellular genes (BLAST E value ≤ e-5) or contained a putative conserved domain as predicted by BLASTp. ORFs were numbered from left to right, with alphabetic sub-ordering used to indicate multiple potential fragments of larger avipoxvirus ORFs.

Promoters described by Afonso et al. [9] were predicted using CLC motif search tool and tandem repeats were identified using TandemRepeatsFinder [70]. Multiple sequence alignments were performed with progressiveMauve [71] and Base-By-Base v2 [72], and dotplots were done in Jdotter [73].

### Phylogenetic analysis

Phylogenetic analyses were performed on representative amino acid sequences of DNA polymerase as well as on the concatenated amino acid sequences of 17 conserved proteins from each ChPV species, as previously described [24]. Amino acid sequences were obtained from Genbank. For the 17 conserved proteins, the individual sequences of each protein were first aligned using MUSCLE [74]. Gblocks.091b [75] was then used to remove any gaps or divergent blocks according to specified criteria [75]. All 17 protein sequences for each virus were then extracted from the Gblocks output and concatenated manually. For the DNA polymerase and concatenated amino acid sequences, multiple sequence alignments were performed with MUSCLE [74]. An additional analysis was performed using the whole genome nucleotide sequences of CNPV, FPVUS, FeP2 and PEPV, which were aligned with ClustalW in Galaxy. Appropriate phylogenetic models were selected using ProtTest 2.4 [76], and phylogenetic analysis was performed in MEGA 5.10 [77] using both maximum parsimony and maximum likelihood methods with 100 bootstrap replicates.

### Nucleotide sequence accession number

The sequences of the South African Feral Pigeonpox virus (FeP2) and Penguinpox virus (PEPV) have been deposited in the NCBI database under GenBank accession numbers: [FeP2 Genbank: KJ801920] and [PEPV Genbank: KJ859677].

## Competing interests

The authors declare that they have no competing interests.

## Authors’ contributions

KO, OC, ND and ALW designed the study. KO and OC performed the experiments. KO, OC and APV performed the bioinformatics and data analysis. KO wrote the inital manuscript. All authors read, edited and approved the final manuscript.

## Supplementary Material

Additional file 1: Table S1 FeP2 and PEPV Open Reading Frames. Click here for file

Additional file 2 Flow diagram depicting the customized bioinformatics pipeline used to analyse FeP2 and PEPV sequencing data. Click here for file
