# Supplementary material for: The complete genome sequences of poxviruses isolated from a penguin and a pigeon in South Africa and comparison to other sequenced avipoxviruses
Source: BMC Genomics. 2014 Jun 12;15:463. doi: 10.1186/1471-2164-15-463 (PMC4229897; doi:10.1186/1471-2164-15-463)
Supplement: Additional file 1: Table S1 — FeP2 and PEPV Open Reading Frames. [file 1471-2164-15-463-S1.docx]

**Additional file 1: Table S1. FeP2 and PEPV Open Reading Frames**

| FEP2 | | | PEPV | | | Homolog ^α^ | Homolog length (aa) | % amino acid identity | | | VACV ORF | Description/ putative function |
| --- | --- | --- | --- | --- | --- | --- | --- | --- | --- | --- | --- | --- |
| FEP2 ORF | length (aa) | FeP2 status | PEPV ORF | length (aa) | PEPV Status |  |  | PEPV vs FeP2 | PEPV vs homolog | FeP2 vs homolog |  |  |
| 1 | 200 | I | 1 | 204 | I | fpv001 | 205 | 87.3 | 77.7 | 77.2 | A40R | C-type lectin family |
| 2 | 66 | F | - | - | a | cnpv021/fpv246 | 1585 | - | - | 5.6 |  | Hypothetical protein Aasi_1435 [Candidatus Amoebophilus asiaticus 5a2] |
| - | - | a | 2 | 164 | F | cnpv319/fpv246 | 739 | - | 8.3 | - |  | Ankyrin repeat family |
| - | - | a | 3 | 115 | F | cnpv310/fpv246 | 537 | - | 8.7 | - |  |  |
| **3** | **222** | **I** | **4** | **222** | **I** | **fpv002** | **222** | **98.2** | **91.0** | **91.0** |  | **Hypothetical protein** |
| 4a | 46 | F | 5 | 46 | F | cnpv006/fpv002.5 | 182 | 100.0 | 14.8 | 14.8 |  | Hypothetical protein |
| 4b | 38 | F | - | - | a | cnpv006/fpv002.5 | 182 | - | - | 9.7 |  | Hypothetical protein |
| 5 | 467 | I | - | - | a | cnpv320/fpv017 | 465 | - | - | 83.2 |  | Ig-like domain protein |
| 6 | 79 | F | 6 | 191 | I | cnpv309/fpv241 | 196 | 30.3 | 33.7 | 14.1 | M1L | Ankyrin repeat family |
| - | - | a | 7 | 139 | I | fpv250 | 140 | - | 80.9 | - |  | Hypothetical protein |
| **7** | **410** | **I** | **8** | **411** | **I** | **fpv006** | **418** | **95.4** | **85.0** | **84.3** | **C10L** | **C4L/C10L-like gene family protein** |
| 8 | 504 | I | 9 | 508 | I | cnpv015/fpv162 | 528 | 96.9 | 42.7 | 42.1 | B4R | Ankyrin repeat family |
| 9 | 40 | F | - | - | a | TVAG_011430 | 732 | - | - | 3.7 |  | Ankyrin repeat family [Trichomonas vaginalis G3] |
| 10 | 680 | I | 10 | 551 | I | fpv244 | 668 | 76.6 | 24.5 | 31.7 |  | Ankyrin repeat family |
| **11** | **355** | **I** | **11** | **349** | **I** | **fpv010** | **355** | **92.4** | **82.8** | **84.5** |  | **Serpin family** |
| **12** | **293** | **I** | **12** | **288** | **I** | **fpv011** | **278** | **91.1** | **74.1** | **74.4** |  | **α-SNAP** |
| 13 | 518 | I | 13 | 518 | I | fpv246 | 592 | 89.6 | 29.4 | 30.2 |  |  |
| 14 | 178 | I | 14 | 174 | I | IL10 (Ficedula albicollis^α^) | 177 | 80.9 | 31.1 | 28.3 |  | Interleukin-10 |
| **15** | **330** | **I** | **15** | **329** | **I** | **fpv012** | **331** | **92.7** | **78.0** | **76.2** |  | **Ankyrin repeat family** |
| 16 | 403 | I | 16 | 402 | I | cnpv028/fpv240 | 362 | 94.8 | 35.7 | 34.9 | B4R | Ankyrin repeat family |
| 17 | 437 | I | 17 | 437 | I | fpv014 | 437 | 95.2 | 86.3 | 86.7 |  | Ankyrin repeat family |
| 18 | 170 | I | 18 | 171 | I | fpv015 | 177 | 84.8 | 74.6 | 75.7 |  | Hypothetical protein |
| **19** | **237** | **I** | **19** | **238** | **I** | **fpv016** | **238** | **85.7** | **80.7** | **79.8** |  | **Hypothetical protein** |
| **20** | **245** | **I** | **20** | **245** | **I** | **fpv017** | **245** | **89.0** | **77.7** | **76.9** |  | **V-type Ig domain** |
| **21** | **683** | **I** | **21** | **683** | **I** | **fpv018** | **700** | **87.6** | **78.0** | **83.9** |  | **Ankyrin repeat family** |
| **22** | **97** | **I** | **22** | **113** | **I** | **fpv019** | **104** | **70.7** | **64.6** | **56.3** |  | **Hypothetical protein** |
| 23 | 189 | I | 23 | 189 | I | cnpv037 | 171 | 94.7 | 43.0 | 44.0 |  | Hypothetical protein (fragment in FPV) |
| **24** | **427** | **I** | **24** | **429** | **I** | **fpv020** | **426** | **93.2** | **85.6** | **87.1** | **C10L** | **C4L/C10L-like family** |
| **25** | **331** | **I** | **25** | **334** | **I** | **fpv021** | **320** | **89.6** | **79.1** | **78.3** |  | **G-protein-coupled receptor family** |
| **26** | **581** | **I** | **26** | **579** | **I** | **fpv022** | **578** | **87.8** | **83.8** | **84.9** |  | **Ankyrin repeat family** |
| **27** | **434** | **I** | **27** | **434** | **I** | **fpv023** | **434** | **97.0** | **91.5** | **90.3** |  | **Ankyrin repeat family** |
| **28** | **594** | **I** | **28** | **595** | **I** | **fpv024** | **596** | **95.3** | **89.6** | **90.0** |  | **Ankyrin repeat family** |
| **29** | **203** | **I** | **29** | **203** | **I** | **fpv025** | **203** | **95.6** | **90.2** | **86.7** |  | **Hypothetical protein** |
| 30 | 498 | I | 30 | 509 | I | cnpv044/fpv024 | 480 | 86.8 | 39.1 | 38.9 | B4R | Ankyrin repeat family |
| 31 | 67 | F | 31 | 406 | I | fpv026 | 436 | 12.6 | 78.5 | 10.8 |  | Ankyrin repeat family |
| - | - | a | 32 | 332 | I | fpv027 | 336 | - | 82.4 | - |  | G-protein-coupled receptor family |
| 32 | 44 | T | 33 | 180 | I | fpv028 | 180 | 14.4 | 88.3 | 15.4 |  | hypothetical protein |
| 33 | 464 | I | - | - | a | cnpv046/fpv024 | 450 | - | - | 48.4 |  | Ankyrin repeat family |
| **34** | **126** | **I** | **34** | **126** | **I** | **fpv029** | **124** | **95.2** | **90.5** | **89.7** |  | **Hypothetical protein** |
| **35** | **808** | **I** | **35** | **815** | **I** | **fpv030** | **817** | **90.1** | **83.4** | **82.6** |  | **Alkaline phosphodiesterase** |
| **36** | **341** | **I** | **36** | **341** | **I** | **fpv031** | **341** | **91.8** | **90.3** | **91.8** |  | **Ankyrin repeat family** |
| - | - | a | 37 | 368 | I | Fp9.032 | 375 | - | 85.6 | - |  | DNAse II |
| - | - | a | 38 | 291 | I | fpv033 | 287 | - | 85.9 | - |  | α-SNAP |
| 37 | 44 | F | 39 | 69 | F | fpv034 | 415 | 5.3 | 6.5 | 7.5 |  | Ankyrin repeat family |
| 38 | 135 | I | 40 | 135 | I | fpv035 | 135 | 97.0 | 96.3 | 96.0 |  | Hypothetical protein |
| **39** | **164** | **I** | **41** | **165** | **I** | **fpv037** | **162** | **89.1** | **67.9** | **68.3** |  | **Hypothetical protein** |
| **40** | **145** | **I** | **42** | **145** | **I** | **fpv038** | **145** | **94.5** | **91.7** | **89.7** | **F2L** | **dUTP pyrophosphatase** |
| **41** | **175** | **I** | **43** | **175** | **I** | **fpv039** | **175** | **91.4** | **80.0** | **81.1** |  | **Bcl-2** |
| **42** | **337** | **I** | **44** | **337** | **I** | **fpv040** | **337** | **96.7** | **92.3** | **91.4** |  | **Serpin family** |
| **43** | **226** | **I** | **45** | **220** | **I** | **fpv041** | **206** | **87.8** | **60.5** | **57.8** |  | **Hypothetical protein** |
| **44** | **564** | **I** | **46** | **564** | **I** | **fpv043** | **564** | **97.5** | **91.1** | **89.9** | **A50R** | **DNA ligase** |
| **45** | **358** | **I** | **47** | **358** | **I** | **fpv044** | **358** | **96.1** | **90.2** | **91.1** |  | **Serpin family** |
| **46** | **370** | **I** | **48** | **370** | **I** | **fpv046** | **370** | **93.2** | **82.8** | **82.8** | **A44L** | **Hydroxysteroid dehydrogenase** |
| **47** | **576** | **I** | **49** | **576** | **I** | **fpv047** | **612** | **94.1** | **74.1** | **74.4** | **A39R** | **Semaphorin** |
| **48** | **261** | **I** | **50** | **261** | **I** | **fpv048** | **261** | **97.7** | **94.6** | **93.9** |  | **GNS1/SUR4** |
| ***49*** | ***154*** | ***I*** | ***51*** | ***154*** | ***I*** | ***fpv049*** | ***154*** | ***96.1*** | ***95.5*** | ***94.8*** | ***A1L*** | ***Late transcription factor VLTF2*** |
| ***50*** | ***552*** | ***I*** | ***52*** | ***552*** | ***I*** | ***fpv050*** | ***552*** | ***98.2*** | ***96.7*** | ***97.1*** | ***D13L*** | ***Rifampicin resistance, N3L protein*** |
| ***51*** | ***289*** | ***I*** | ***53*** | ***289*** | ***I*** | ***fpv051*** | ***289*** | ***98.6*** | ***94.5*** | ***95.2*** | ***D12L*** | ***mRNA capping enzyme*** |
| ***52*** | ***637*** | ***I*** | ***54*** | ***577*** | ***I*** | ***fpv052*** | ***637*** | ***89.2*** | ***86.7*** | ***95.6*** | ***D11L*** | ***NPH-1 transcription termination factor*** |
| ***53*** | ***225*** | ***I*** | ***55*** | ***225*** | ***I*** | ***fpv053*** | ***225*** | ***96.9*** | ***95.1*** | ***95.6*** | ***D10L*** | ***muT motif; gene expression regulation*** |
| ***54*** | ***237*** | ***I*** | ***56*** | ***236*** | ***I*** | ***fpv054*** | ***231*** | ***97.5*** | ***92.4*** | ***92.4*** | ***D9R*** | ***muT motif*** |
| 55 | 274 | I | 57 | 274 | I | fpv055 | 275 | 86.5 | 63.8 | 64.1 |  | V-type Ig Domain |
| ***56*** | ***161*** | ***I*** | ***58*** | ***161*** | ***I*** | ***fpv056*** | ***161*** | ***98.1*** | ***96.3*** | ***96.9*** | ***D7R*** | ***RNA polymerase subunit RPO18*** |
| ***57*** | ***633*** | ***I*** | ***59*** | ***633*** | ***I*** | ***fpv057*** | ***633*** | ***99.1*** | ***98.6*** | ***98.6*** | ***D6R*** | ***Early transcription factor VETFs*** |
| ***58*** | ***791*** | ***I*** | ***60*** | ***791*** | ***I*** | ***fpv058*** | ***791*** | ***99.1*** | ***97.9*** | ***98.0*** | ***D5R*** | ***NTPase; DNA replication*** |
| - | - | a | 61 | 219 | I | fpv059 | 219 | - | 86.3 | - |  | Deoxycytidine kinase |
| 59 | 200 | I | 62 | 35 | F | fpv060 | 188 | 12.5 | 14.9 | 73.5 |  | CC chemokine family |
| 60 | 109 | I | 63 | 112 | I | fpv061 | 129 | 80.7 | 68.5 | 66.4 |  | CC chemokine family |
| 61 | 199 | I | - | - | a | cnpv232/fpv060 | 204 | - | - | 30.2 |  | CC chemokine family |
| ***62*** | ***218*** | ***I*** | ***64*** | ***218*** | ***I*** | ***fpv062*** | ***218*** | ***96.3*** | ***97.7*** | ***97.7*** | ***D4R*** | ***Uracil DNA glycosylase*** |
| 63a | 93 | T | 65 | 401 | I | fpv063 | 400 | 41.4 | 79.4 | 39.9 |  |  |
| 63b | 134 | T | - | - | a | fpv063 | 400 | 25.4 | - | 21.8 |  |  |
| 64 | 134 | F | 66a | 42 | T | fpv064 | 200 |  | 18.0 | 61.2 |  | Glutathione peroxidase |
| - | - | - | 66b | 134 | T | fpv064 |  | 97.8 | 62.7 | 61.2 |  |  |
| **65** | **110** | **I** | **67** | **110** | **I** | **fpv065** | **111** | **94.6** | **78.4** | **80.2** |  | **Hypothetical protein** |
| **66** | **138** | **I** | **68** | **137** | **I** | **fpv066** | **122** | **92.0** | **73.7** | **73.2** |  | **Hypothetical protein** |
| **67** | **93** | **I** | **69** | **85** | **I** | **fpv067** | **90** | **90.3** | **86.8** | **87.1** |  | **HT motif family** |
| **68** | **131** | **I** | **70** | **143** | **I** | **fpv068** | **133** | **74.8** | **52.6** | **66.2** |  | **Hypothetical protein** |
| ***69*** | ***268*** | ***I*** | ***71*** | ***269*** | ***I*** | ***fpv069*** | ***270*** | ***97.0*** | ***92.6*** | ***92.6*** | ***D3R*** | ***Virion protein*** |
| **70** | **273** | **I** | **72** | **273** | **I** | **fpv070** | **273** | **94.5** | **84.6** | **85.0** |  | **T10 gene product** |
| 71 | 43 | I | 73 | 43 | I | cnpv095/fpv070.5 | 45 | 93.0 | 51.1 | 53.3 |  | Hypothetical protein |
| - | - | a | 74 | 77 | T | UBIQUITIN | 98 | - | 76.5 | - |  | Ubiquitin |
| **72** | **287** | **I** | **75** | **287** | **I** | **fpv071** | **289** | **92.3** | **88.9** | **88.9** |  | **Hypothetical protein** |
| 73 | 54 | F | - | - | a | cnpv098 | 80 | - | - | 27.7 |  | Hypothetical protein |
| 74 | 82 | F | - | - | a | fpv072 | 186 | - | - | 39.3 |  | Beta-Nerve growth factor |
| 75 | 106 | T | - | - | a | fpv073 | 174 | - | - | 37.9 |  | Interleukin (IL18) binding protein |
| - | - | a | 76 | 130 | F | cnpv012/fpv229 | 189 | - | 22.6 | - |  | Hypothetical protein |
| **76** | **101** | **I** | **77** | **104** | **I** | **fpv074** | **104** | **85.6** | **78.1** | **77.9** |  | **Hypothetical protein** |
| **77** | **187** | **I** | **78** | **211** | **I** | **fpv075** | **199** | **84.8** | **74.9** | **82.4** |  | **N1R/p28 family** |
| - | - | a | 79 | 51 | F | fpv076 | 144 | - | 22.22 | - |  | Beta-Nerve growth factor |
| ***78*** | ***125*** | ***I*** | ***80*** | ***125*** | ***I*** | ***fpv077*** | ***125*** | ***100.0*** | ***95.2*** | ***95.2*** | ***G4L*** | ***Gutaredoxin*** |
| ***79*** | ***225*** | ***I*** | ***81*** | ***225*** | ***I*** | ***fpv079*** | ***225*** | ***96.9*** | ***93.3*** | ***94.2*** | ***G2R*** | ***Putative elongation factor*** |
| ***80*** | ***103*** | ***I*** | ***82*** | ***103*** | ***I*** | ***fpv078*** | ***103*** | ***98.1*** | ***94.2*** | ***92.2*** | ***G3L*** | ***Hypothetical protein*** |
| **81** | **335** | **I** | **83** | **336** | **I** | **fpv080** | **363** | **92.3** | **68.9** | **69.0** |  | **Transforming Growth Factor β (TGF-B)** |
| ***82*** | ***627*** | ***I*** | ***84*** | ***624*** | ***I*** | ***fpv081*** | ***626*** | ***97.3*** | ***95.1*** | ***94.4*** | ***G1L*** | ***Metalloprotease*** |
| ***83*** | ***682*** | ***I*** | ***85*** | ***682*** | ***I*** | ***fpv082*** | ***682*** | ***96.5*** | ***94.1*** | ***93.8*** | ***I8R*** | ***DNA/RNA helicase/NPH-11*** |
| ***84*** | ***421*** | ***I*** | ***86*** | ***421*** | ***I*** | ***fpv083*** | ***421*** | ***98.1*** | ***96.2*** | ***96.7*** | ***I7L*** | ***Virion core proteinase*** |
| ***85*** | ***391*** | ***I*** | ***87*** | ***390*** | ***I*** | ***fpv084*** | ***390*** | ***98.7*** | ***94.4*** | ***94.4*** | ***I6L*** | ***DNA-binding protein*** |
| ***86*** | ***81*** | ***I*** | ***88*** | ***81*** | ***I*** | ***fpv085*** | ***81*** | ***100.0*** | ***90.1*** | ***90.1*** | ***I5L*** | ***IMV membrane protein*** |
| **87** | **183** | **I** | **89** | **183** | **I** | **fpv086** | **183** | **95.6** | **89.1** | **86.9** | **J2R** | **Thymidine kinase** |
| **88** | **82** | **I** | **90** | **91** | **I** | **fpv087** | **91** | **93.4** | **92.3** | **92.3** |  | **HT motif family** |
| ***89*** | ***291*** | ***I*** | ***91*** | ***290*** | ***I*** | ***fpv088*** | ***290*** | ***96.6*** | ***95.5*** | ***94.9*** | ***I3L*** | ***DNA binding phosphoprotein*** |
| ***90*** | ***65*** | ***I*** | ***92*** | ***65*** | ***I*** | ***fpv089*** | ***65*** | ***96.9*** | ***95.4*** | ***95.4*** | ***I2L*** | ***Hypothetical protein*** |
| ***91*** | ***311*** | ***I*** | ***93*** | ***311*** | ***I*** | ***fpv090*** | ***311*** | ***99.4*** | ***98.4*** | ***98.4*** | ***I1L*** | ***Virion protein*** |
| 92 | 34 | I | 94 | 34 | I | fpO3L | 34 | 91.2 | 91.2 | 88.2 | O3L | Orthologue of vaccinia O3L & MC043.1L |
| **93** | **656** | **I** | **95** | **656** | **I** | **fpv091** | **656** | **96.2** | **88.3** | **89.5** | **O1L** | **Hypothetical protein** |
| **94** | **131** | **I** | **96** | **131** | **I** | **fpv092** | **131** | **95.4** | **93.9** | **93.1** | **E11L** | **Hypothetical protein** |
| ***95*** | ***94*** | ***I*** | ***97*** | ***94*** | ***I*** | ***fpv093*** | ***94*** | ***95.7*** | ***90.4*** | ***92.6*** | ***E10R*** | ***Sulfhydryl oxidase ERV1*** |
| ***96*** | ***988*** | ***I*** | ***98*** | ***988*** | ***I*** | ***fpv094*** | ***988*** | ***98.5*** | ***92.9*** | ***92.4*** | ***E9L*** | ***DNA polymerase*** |
| ***97*** | ***282*** | ***I*** | ***99*** | ***280*** | ***I*** | ***fpv095*** | ***272*** | ***98.6*** | ***89.3*** | ***89.4*** | ***E8R*** | ***Hypothetical protein*** |
| ***98*** | ***571*** | ***I*** | ***100*** | ***571*** | ***I*** | ***fpv096*** | ***571*** | ***99.1*** | ***96.2*** | ***95.8*** | ***E6R*** | ***Hypothetical protein*** |
| 99 | 1885 | I | 101 | 1894 | I | fpv097 | 1912 | 94.3 | 86.2 | 85.5 |  | VARV B22R family |
| **100** | **1826** | **I** | **102** | **1812** | **I** | **fpv098** | **1802** | **93.4** | **86.7** | **95.5** |  | **VARV B22R family** |
| **101** | **1937** | **I** | **103** | **1922** | **I** | **fpv099** | **1949** | **91.3** | **84.9** | **84.7** |  | **VARV B22R family** |
| ***102*** | ***182*** | ***I*** | ***104*** | ***182*** | ***I*** | ***fpv100*** | ***182*** | ***97.8*** | ***97.8*** | ***96.7*** | ***E4L*** | ***RNA pol subunit RPO30*** |
| ***103*** | ***717*** | ***I*** | ***105*** | ***717*** | ***I*** | ***fpv101*** | ***717*** | ***95.5*** | ***93.4*** | ***92.3*** | ***E2L*** | ***Hypothetical protein*** |
| ***104*** | ***472*** | ***I*** | ***106*** | ***472*** | ***I*** | ***fpv102*** | ***472*** | ***98.9*** | ***97.5*** | ***98.1*** | ***E1L*** | ***Poly(A) polymerase large subunit, PAP-L*** |
| ***105*** | ***114*** | ***I*** | ***107*** | ***114*** | ***I*** | ***fpv103*** | ***114*** | ***100.0*** | ***100.0*** | ***100.0*** | ***F17R*** | ***DNA binding virion core phosphoprotein*** |
| **106** | **210** | **I** | **108** | **210** | **I** | **fpv104** | **210** | **95.2** | **81.4** | **81.4** |  | **Hypothetical protein** |
| ***107*** | ***149*** | ***I*** | ***109*** | ***149*** | ***I*** | ***fpv105*** | ***148*** | ***99.3*** | ***96.6*** | ***96.0*** | ***F15L*** | ***Hypothetical protein*** |
| **108** | **99** | **I** | **110** | **99** | **I** | **fpv106** | **99** | **91.9** | **65.0** | **66.0** |  | **Conserved hypothetical protein** |
| **109** | **1780** | **I** | **111** | **1779** | **I** | **fpv107** | **1777** | **95.1** | **85.4** | **85.4** |  | **VARV B22R family** |
| ***110*** | ***377*** | ***I*** | ***112*** | ***377*** | ***I*** | ***fpv108*** | ***377*** | ***96.6*** | ***94.2*** | ***94.7*** | ***F13L*** | ***Virion envelope protein*** |
| ***111*** | ***639*** | ***I*** | ***113*** | ***639*** | ***I*** | ***fpv109*** | ***630*** | ***93.7*** | ***85.5*** | ***85.0*** | ***F12L*** | ***Virion release protein*** |
| **112** | **142** | **I** | **114** | **452** | **I** | **fpv110** | **451** | **83.4** | **84.7** | **75.0** | **F11L** |  |
| ***113*** | ***444*** | ***I*** | ***115*** | ***443*** | ***I*** | ***fpv111*** | ***444*** | ***97.8*** | ***96.4*** | ***96.9*** | ***F10L*** | ***SER/THR protein kinase (virus assembly)*** |
| ***114*** | ***213*** | ***I*** | ***116*** | ***213*** | ***I*** | ***fpv112*** | ***213*** | ***96.2*** | ***93.0*** | ***94.4*** | ***F9L*** | ***Hypothetical protein*** |
| **115** | **66** | **I** | **117** | **66** | **I** | **fpv113** | **66** | **97.0** | **98.5** | **95.5** |  |  |
| **116** | **183** | **I** | **118** | **183** | **I** | **fpv114** | **183** | **95.1** | **95.6** | **92.4** |  | **HAL3 domain** |
| 117 | 143 | F | - | - | a | cnpv011/fpv246 | 586 | - | - | 8.5 |  | Ankyrin repeat family |
| 118 | 60 | F | 119 | 84 | F | cnpv004/fpv246 | 514 | 53.6 | 8.0 | 5.3 |  |  |
| - | - | a | 120 | 66 | F | Neosartorya fischeri NRRL 181 | 1174 | - | 1.8 | - |  | Pfs, NACHT and Ankyrin domain protein |
| - | - | a | 121 | 541 | I | fpv115 | 542 | - | 78.0 | - |  | Ankyrin repeat family |
| 119 | 122 | I | 122 | 82 | F | fpv116 | 120 | 63.9 | 56.7 | 81.2 |  | CC-chemokine family |
| ***120*** | ***440*** | ***I*** | ***123*** | ***440*** | ***I*** | ***fpv117*** | ***440*** | ***96.4*** | ***90.9*** | ***91.6*** | ***G5R*** | ***Hypothetical protein*** |
| ***121*** | ***63*** | ***I*** | ***124*** | ***63*** | ***I*** | ***fpv118*** | ***63*** | ***98.4*** | ***98.4*** | ***96.8*** | ***G5.5R*** | ***RNA pol subunit RPO7*** |
| ***122*** | ***188*** | ***I*** | ***125*** | ***188*** | ***I*** | ***fpv119*** | ***188*** | ***95.7*** | ***99.5*** | ***95.2*** | ***G6R*** | ***Hypothetical protein*** |
| ***123*** | ***343*** | ***I*** | ***126*** | ***343*** | ***I*** | ***fpv120*** | ***343*** | ***98.3*** | ***95.9*** | ***96.5*** | ***G7L*** | ***Virion core protein*** |
| - | - | a | 127 | 83 | T | cnpv221/fpv124 | 281 | - | 26.0 | - |  | N1R/p28 family |
| - | - | a | 128 | 148 | F | cnpv165/fpv124 | 346 | - | 35.8 | - |  | N1R/p28 family |
| - | - | a | 129a | 1442 | T | fpv122 | 1870 | - | 51.6 | - |  | VARV B22R family |
| - | - | a | 129b | 501 | T | fpv122 | 1870 | - | 22.9 | - |  | VARV B22R family |
| - | - | a | 130 | 1771 | I | fpv123 | 1766 | - | 77.0 | - |  | VARV B22R family |
| - | - | a | 131 | 62 | F | cnpv162/fpv080 | 149 | - | 29.5 | - |  | Transforming Growth Factor (TGF)-beta-like protein |
| - | - | a | 132 | 116 | I | cnpv086 | 117 | - | 46.2 | - |  | Tumor Necrosis Factor Receptor (TNFR)-like protein |
| 124 | 203 | I | - | - | a | cnpv012/fpv229 | 189 | - | - | 31.1 |  | Hypothetical protein |
| 125 | 239 | I | - | - | a | cnpv224 | 239 | - | - | 55.7 |  | hypothetical protein |
| 126 | 211 | I | 133 | 217 | I | cnpv170 | 212 | 90.8 | 80.2 | 81.6 |  | Thymidylate Kinase |
| ***127*** | ***260*** | ***I*** | ***134*** | ***260*** | ***I*** | ***fpv126*** | ***260*** | ***99.2*** | ***99.2*** | ***98.9*** | ***G8R*** | ***VLTF-1*** |
| ***128*** | ***336*** | ***I*** | ***135*** | ***322*** | ***I*** | ***fpv127*** | ***336*** | ***97.0*** | ***93.8*** | ***92.6*** | ***G9R*** | ***Myristylated protein*** |
| ***129*** | ***243*** | ***I*** | ***136*** | ***243*** | ***I*** | ***fpv128*** | ***243*** | ***98.8*** | ***97.1*** | ***95.9*** | ***L1R*** | ***Myristylated protein*** |
| ***130*** | ***96*** | ***I*** | ***137*** | ***96*** | ***I*** | ***fpv129*** | ***96*** | ***96.9*** | ***87.5*** | ***86.5*** | ***L2R*** | ***Hypothetical protein*** |
| ***131*** | ***301*** | ***I*** | ***138*** | ***301*** | ***I*** | ***fpv130*** | ***301*** | ***98.7*** | ***93.7*** | ***93.7*** | ***L3L*** | ***Hypothetical protein*** |
| ***132*** | ***253*** | ***I*** | ***139*** | ***253*** | ***I*** | ***fpv131*** | ***253*** | ***98.4*** | ***94.1*** | ***94.5*** | ***L4R*** | ***DNA binding virion core VP8*** |
| ***133*** | ***129*** | ***I*** | ***140*** | ***129*** | ***I*** | ***fpv132*** | ***129*** | ***96.9*** | ***93.8*** | ***93.8*** | ***L5R*** | ***Putative membrane protein*** |
| ***134*** | ***148*** | ***I*** | ***141*** | ***148*** | ***I*** | ***fpv133*** | ***148*** | ***98.7*** | ***96.6*** | ***96.6*** | ***J1R*** | ***Hypothetical protein*** |
| ***135*** | ***308*** | ***I*** | ***142*** | ***308*** | ***I*** | ***fpv134*** | ***308*** | ***97.4*** | ***95.8*** | ***94.8*** | ***J3R*** | ***PolyA polymerase (PAPs)*** |
| ***136*** | ***186*** | ***I*** | ***143*** | ***186*** | ***I*** | ***fpv135*** | ***186*** | ***97.9*** | ***95.2*** | ***94.6*** | ***J4R*** | ***RNA pol Subunit RPO22*** |
| ***137*** | ***137*** | ***I*** | ***144*** | ***137*** | ***I*** | ***fpv136*** | ***137*** | ***94.9*** | ***90.5*** | ***91.2*** | ***J5L*** | ***Membrane protein*** |
| ***138*** | ***1287*** | ***I*** | ***145*** | ***1287*** | ***I*** | ***fpv137*** | ***1287*** | ***98.5*** | ***97.6*** | ***96.8*** | ***J6R*** | ***RNA pol Subunit RPO147*** |
| ***139*** | ***166*** | ***I*** | ***146*** | ***166*** | ***I*** | ***fpv138*** | ***166*** | ***99.4*** | ***95.2*** | ***94.6*** | ***H1L*** | ***Protein tyrosine Phosphatase*** |
| ***140*** | ***190*** | ***I*** | ***147*** | ***190*** | ***I*** | ***fpv139*** | ***190*** | ***99.0*** | ***97.4*** | ***96.8*** | ***H2R*** | ***Hypothetical protein*** |
| ***141*** | ***333*** | ***I*** | ***148*** | ***333*** | ***I*** | ***fpv140*** | ***327*** | ***94.6*** | ***87.4*** | ***87.4*** | ***H3L*** | ***Virion env protein(p35)*** |
| ***142*** | ***799*** | ***I*** | ***149*** | ***799*** | ***I*** | ***fpv141*** | ***798*** | ***98.1*** | ***96.6*** | ***96.4*** | ***H4L*** | ***RNA polymerase associated protein RAP94*** |
| ***143*** | ***174*** | ***I*** | ***150*** | ***174*** | ***I*** | ***fpv142*** | ***174*** | ***94.8*** | ***90.2*** | ***89.7*** | ***H5R*** | ***VLTF-4*** |
| ***144*** | ***316*** | ***I*** | ***151*** | ***316*** | ***I*** | ***fpv143*** | ***316*** | ***98.7*** | ***97.2*** | ***96.2*** | ***H6R*** | ***DNA topoisomerase*** |
| ***145*** | ***152*** | ***I*** | ***152*** | ***152*** | ***I*** | ***fpv144*** | ***152*** | ***99.34*** | ***92.8*** | ***92.1*** | ***H7R*** | ***Putative 17 kDa protein*** |
| **146** | **103** | **I** | **153** | **103** | **I** | **fpv145** | **103** | **91.3** | **87.38** | **84.47** |  |  |
| ***147*** | ***852*** | ***I*** | ***154*** | ***822*** | ***I*** | ***fpv146*** | ***851*** | ***97.7*** | ***94.5*** | ***94.0*** | ***D1R*** | ***mRNA capping enzyme, large subunit*** |
| 148 | 63 | F | 155 | 84 | I | fpv147 | 104 | 66.7 | 61.5 | 51.9 |  | HT motif family |
| ***149*** | ***140*** | ***I*** | ***156*** | ***140*** | ***I*** | ***fpv148*** | ***139*** | ***97.1*** | ***90.7*** | ***90.0*** | ***D2L*** | ***virion protein*** |
| 150 | 45 | F | - | - | a | Erysipelotrichaceae bacterium 3_1_53 | 112 | - | - | 16.0 |  | Hypothetical protein [Erysipelotrichaceae bacterium 3_1_53] |
| **151** | **190** | **I** | **157** | **189** | **I** | **fpv149** | **186** | **97.4** | **85.7** | **84.7** |  | **Hypothetical protein** |
| **152** | **284** | **I** | **158** | **283** | **I** | **fpv150** | **276** | **89.5** | **82.9** | **81.7** |  | **N1R/p28 gene family protein** |
| **153** | **238** | **I** | **159** | **238** | **I** | **fpv151** | **235** | **91.2** | **83.7** | **84.1** |  | **dCK** |
| - | - | a | 160 | 127 | I | fpv152 | 127 | - | 81.1 | - |  | HT motif family |
| 154 | 209 | I | - | - | a | fpv153 | 208 | - | - | 77.3 |  | Hypothetical protein |
| - | - | a | 161 | 270 | E | fpv154 | 150 | - | 41.2 | - |  | Hypothetical protein |
| **155** | **410** | **I** | **162** | **410** | **I** | **fpv155** | **408** | **91.0** | **85.9** | **85.9** |  | **N1R/p28 family** |
| 156 | 132 | I | 163a | 51 | T | fpv156 | 132 | 33.3 | 27.3 | 87.9 |  | HT motif family |
| - | - | - | 163b | 96 | T | fpv156 |  | 64.4 | 60.6 | - |  |  |
| **157** | **327** | **I** | **164** | **329** | **I** | **fpv157** | **311** | **93.0** | **79.6** | **78.4** |  | **N1R/p28 family** |
| **158** | **464** | **I** | **165** | **464** | **I** | **fpv158** | **464** | **95.3** | **93.3** | **95.0** |  | **Photolyase** |
| 159 | 246 | I | 166 | 246 | I | fpv159 | 241 | 94.7 | 84.6 | 83.7 |  | N1R/p28 |
| 160 | 156 | I | 167 | 156 | I | fpv160 | 156 | 97.4 | 94.2 | 94.2 |  | Hypothetical protein |
| **161** | **149** | **I** | **168** | **149** | **I** | **fpv161** | **157** | **95.3** | **82.8** | **83.4** |  | **N1R/p28 family** |
| 162 | 133 | I | 169 | 133 | I | cnpv210/fpv075 | 131 | 95.5 | 43.0 | 43.7 |  | N1R/p28 family |
| 163 | 45 | I | 170 | 45 | I | cnpv211/fpv161.5 | 54 | 95.6 | 32.7 | 36.4 |  | Hypothetical protein |
| 164 | 160 | I | 171 | 160 | I | cnpv212/fpv124 | 176 | 96.3 | 52.8 | 53.4 |  | N1R/p28 family |
| **165** | **591** | **I** | **172** | **603** | **I** | **fpv162** | **603** | **85.9** | **81.1** | **78.3** |  | **Ankyrin repeat family** |
| 166 | 256 | I | 173 | 256 | I | fpv163 | 263 | 88.7 | 77.2 | 76.1 |  | N1R/p28 family |
| 167 | 46 | F | 174 | 741 | I | fpv162 | 603 | 5.9 | 28.4 | 3.3 |  | Ankyrin repeat family |
| **168** | **383** | **I** | **175** | **386** | **I** | **fpv164** | **383** | **85.4** | **65.5** | **67.4** |  | **Hypothetical protein** |
| - | - | a | 176 | 150 | F | cnpv041/fpv131 | 430 | - | 13.0 | - |  | Ankyrin repeat family |
| ***169*** | ***225*** | ***I*** | ***177*** | ***225*** | ***I*** | ***fpv165*** | ***225*** | ***99.6*** | ***94.7*** | ***95.1*** | ***A2L*** | ***Late transcription factor VLTF-3*** |
| ***170*** | ***72*** | ***I*** | ***178*** | ***72*** | ***I*** | ***fpv166*** | ***72*** | ***100.0*** | ***95.8*** | ***95.8*** | ***A2.5L*** | ***Virus redox protein*** |
| ***171*** | ***658*** | ***I*** | ***179*** | ***658*** | ***I*** | ***fpv167*** | ***658*** | ***99.7*** | ***98.6*** | ***98.3*** | ***A3L*** | ***Virion core protein P4b*** |
| ***172*** | ***244*** | ***I*** | ***180*** | ***255*** | ***I*** | ***fpv168*** | ***288*** | ***87.2*** | ***67.7*** | ***63.1*** | ***A4L*** | ***Immunodominant virion protein*** |
| ***173*** | ***169*** | ***I*** | ***181*** | ***169*** | ***I*** | ***fpv169*** | ***167*** | ***98.8*** | ***97.0*** | ***97.0*** | ***A5R*** | ***RNA pol subunit RP019*** |
| ***174*** | ***374*** | ***I*** | ***182*** | ***374*** | ***I*** | ***fpv170*** | ***375*** | ***98.1*** | ***94.1*** | ***94.4*** | ***A6L*** | ***Hypothetical protein*** |
| ***175*** | ***709*** | ***I*** | ***183*** | ***709*** | ***I*** | ***fpv171*** | ***709*** | ***98.7*** | ***97.3*** | ***97.2*** | ***A7L*** | ***Early transcription factor large subunit, VETF-L*** |
| ***176*** | ***301*** | ***I*** | ***184*** | ***301*** | ***I*** | ***fpv172*** | ***301*** | ***99.0*** | ***98.0*** | ***97.7*** | ***A8R*** | ***Intermediate transcription factor VITF-3*** |
| ***177*** | ***76*** | ***I*** | ***185*** | ***76*** | ***I*** | ***fpv173*** | ***76*** | ***94.7*** | ***96.1*** | ***98.7*** | ***A9L*** | ***Hypothetical protein*** |
| ***178*** | ***891*** | ***I*** | ***186*** | ***891*** | ***I*** | ***fpv174*** | ***891*** | ***98.3*** | ***96.0*** | ***96.1*** | ***A10L*** | ***Virion core protein P4a*** |
| ***179*** | ***272*** | ***I*** | ***187*** | ***272*** | ***I*** | ***fpv175*** | ***274*** | ***99.6*** | ***96.7*** | ***96.4*** | ***A11R*** | ***Hypothetical protein*** |
| ***180*** | ***175*** | ***I*** | ***188*** | ***174*** | ***I*** | ***fpv176*** | ***171*** | ***96.6*** | ***90.8*** | ***88.0*** | ***A12L*** | ***Virion protein*** |
| 181 | 49 | T | 189 | 70 | I | fpv177 | 68 | 64.3 | 82.9 | 61.8 |  | Hypothetical protein |
| ***182*** | ***71*** | ***I*** | ***190*** | ***71*** | ***I*** | ***fpv178*** | ***71*** | ***93.0*** | ***84.5*** | ***88.7*** | ***A13L*** | ***Virion protein*** |
| ***183*** | ***91*** | ***I*** | ***191*** | ***91*** | ***I*** | ***fpv179*** | ***91*** | ***98.9*** | ***95.6*** | ***94.5*** | ***A14L*** | ***Virion envelope protein*** |
| ***184*** | ***53*** | ***I*** | ***192*** | ***53*** | ***I*** | ***fpv179.1*** | ***54*** | ***100.0*** | ***98.1*** | ***98.1*** | ***a14.5*** | ***Virion envelope protein*** |
| ***185*** | ***97*** | ***I*** | ***193*** | ***97*** | ***I*** | ***fpv180*** | ***97*** | ***96.9*** | ***95.9*** | ***96.9*** | ***A15L*** | ***Hypothetical protein*** |
| ***186*** | ***369*** | ***I*** | ***194*** | ***369*** | ***I*** | ***fpv181*** | ***369*** | ***95.7*** | ***92.4*** | ***91.9*** | ***A16L*** | ***Putative mystirilated membrane protein*** |
| ***187*** | ***198*** | ***I*** | ***195*** | ***198*** | ***I*** | ***fpv182*** | ***198*** | ***98.5*** | ***97.5*** | ***98.0*** | ***A17L*** | ***Phosphorylated virion membrane protein*** |
| ***188*** | ***462*** | ***I*** | ***196*** | ***462*** | ***I*** | ***fpv183*** | ***462*** | ***97.6*** | ***96.5*** | ***95.9*** | ***A18R*** | ***DNA helicase (transcription elongation)*** |
| ***189*** | ***88*** | ***I*** | ***197*** | ***88*** | ***I*** | ***fpv184*** | ***88*** | ***97.7*** | ***95.5*** | ***93.2*** | ***A19L*** | ***Hypothetical protein*** |
| ***190*** | ***113*** | ***I*** | ***198*** | ***113*** | ***I*** | ***fpv186*** | ***113*** | ***92.0*** | ***92.0*** | ***92.0*** | ***A21L*** | ***Hypothetical protein*** |
| ***191*** | ***432*** | ***I*** | ***199*** | ***432*** | ***I*** | ***fpv185*** | ***433*** | ***97.9*** | ***94.5*** | ***92.8*** | ***A20R*** | ***Processivity factor*** |
| ***192*** | ***156*** | ***I*** | ***200*** | ***161*** | ***I*** | ***fpv187*** | ***156*** | ***95.0*** | ***88.8*** | ***91.0*** | ***A22R*** | ***Hypothetical protein*** |
| ***193*** | ***383*** | ***I*** | ***201*** | ***383*** | ***I*** | ***fpv188*** | ***383*** | ***96.3*** | ***94.8*** | ***94.3*** | ***A23R*** | ***Intermediate transcription factor VITF-3*** |
| ***194*** | ***1157*** | ***I*** | ***202*** | ***1157*** | ***I*** | ***fpv189*** | ***1161*** | ***99.2*** | ***97.9*** | ***97.8*** | ***A24R*** | ***RNA pol subunit RPO132*** |
| ***195*** | ***608*** | ***I*** | ***203*** | ***610*** | ***I*** | ***fpv190*** | ***620*** | ***98.0*** | ***87.2*** | ***87.2*** | ***A25L*** | ***A-type inclusion protein*** |
| ***196*** | ***472*** | ***I*** | ***204*** | ***472*** | ***I*** | ***fpv191*** | ***474*** | ***98.7*** | ***92.2*** | ***91.8*** | ***A26L*** | ***A-type inclusion protein*** |
| ***197*** | ***140*** | ***I*** | ***205*** | ***140*** | ***I*** | ***fpv192*** | ***141*** | ***100.0*** | ***95.7*** | ***95.7*** | ***A28L*** | ***Hypothetical protein*** |
| ***198*** | ***302*** | ***I*** | ***206*** | ***302*** | ***I*** | ***fpv193*** | ***302*** | ***97.7*** | ***90.4*** | ***89.4*** | ***A29L*** | ***RNA pol subunit RPO35*** |
| ***199*** | ***74*** | ***I*** | ***207*** | ***74*** | ***I*** | ***fpv194*** | ***74*** | ***98.7*** | ***96.0*** | ***97.3*** | ***A30L*** | ***Hypothetical protein*** |
| 200 | 37 | I | 208 | 38 | I | fp9.194.1 |  | 94.7 | 84.2 | 81.6 |  | A30.5L orthologue |
| **201** | **113** | **I** | **209** | **113** | **I** | **fpv195** | **113** | **97.4** | **90.3** | **91.2** | **A31R** | **Hypothetical protein** |
| **202** | **120** | **I** | **210** | **120** | **I** | **fpv196** | **120** | **97.5** | **80.8** | **81.7** |  | **Hypothetical protein** |
| ***203*** | ***304*** | ***I*** | ***211*** | ***304*** | ***I*** | ***fpv197*** | ***301*** | ***98.4*** | ***93.1*** | ***92.1*** | ***A32L*** | ***Virion assembly protein*** |
| ***204*** | ***173*** | ***I*** | ***212*** | ***151*** | ***I*** | ***fpv198*** | ***173*** | ***97.7*** | ***91.9*** | ***92.5*** | ***A34R*** | ***C-type lectin-like protein*** |
| 205 | 106 | F | 213 | 220 | I | fpv199 | 219 | 45.5 | 79.1 | 38.8 |  | V-type Ig Domain |
| 206a | 39 | F | 214 | 263 | I | fpv200 | 265 | 12.6 | 82.6 | 10.9 |  | V-type Ig domain |
| 206b | 58 | F | - | - | - | fpv200 | 265 | 20.5 | - | 17.4 |  | V-type Ig domain |
| **207** | **277** | **I** | **215** | **276** | **I** | **fpv201** | **283** | **94.6** | **85.9** | **87.3** |  | **Hypothetical protein** |
| **208** | **285** | **I** | **216** | **285** | **I** | **fpv203** | **285** | **96.8** | **83.2** | **83.5** |  | **Tyrosine protein kinase** |
| **209** | **342** | **I** | **217** | **342** | **I** | **fpv204** | **342** | **95.3** | **92.4** | **90.6** |  | **Serpin family** |
| **210** | **220** | **I** | **218** | **220** | **I** | **fpv205** | **218** | **98.6** | **82.7** | **82.3** |  | **Hypothetical protein** |
| **211** | **308** | **I** | **219** | **308** | **I** | **fpv206** | **308** | **98.4** | **88.3** | **88.0** |  | **G-protein-coupled receptor family** |
| **212** | **92** | **I** | **220** | **92** | **I** | **fpv207** | **100** | **92.4** | **79.0** | **77.0** |  | **Hypothetical protein** |
| 213 | 67 | T | 221 | 150 | F | CNPV279 | 169 | 20.1 | 45.8 | 13.6 |  | Beta-NGF-like protein |
| **214** | **213** | **I** | **222** | **193** | **I** | **fpv208 (cnpv281)** | **214** | **96.4** | **41.1** | **40.2** |  | **Hypothetical protein** |
| 215 | 139 | I | 223 | 137 | I | fpv209 | 130 | 79.3 | 72.1 | 74.6 |  | HT motif family |
| - | - | a | 224 | 107 | F | cnpv 283 | 111 | - | 48.7 | - |  | CC chemokine family |
| **216** | **123** | **I** | **225** | **123** | **I** | **fpv211** | **125** | **92.7** | **79.4** | **81.0** | **C11R** | **Epidermal Growth Factor-like protein** |
| **217** | **303** | **I** | **226** | **304** | **I** | **fpv212** | **303** | **87.8** | **82.2** | **93.0** | **B1R** | **Serine/threonine protein kinase** |
| **218** | **162** | **I** | **227** | **162** | **I** | **fpv213** | **162** | **95.1** | **90.7** | **90.7** |  | **Hypothetical protein** |
| **219** | **125** | **I** | **228** | **124** | **I** | **fpv214** | **124** | **85.6** | **79.2** | **77.0** |  | **Putative 13.7 kDa protein** |
| **220** | **74** | **I** | **229** | **74** | **I** | **fpv215** | **74** | **100.0** | **94.6** | **94.6** |  | **Hypothetical protein** |
| 221 | 173 | F | - | - | a | LOC100486298 | 959 | - | - | 4.7 |  | Hypothetical protein LOC100486298 [Xenopus (Silurana) tropicalis] |
| **222** | **294** | **I** | **230** | **294** | **I** | **fpv216** | **296** | **92.9** | **86.2** | **84.5** |  | **Ankyrin repeat family** |
| 223 | 143 | I | 231 | 143 | I | Tanapox 67R | 178 | 96.5 | 27.1 | 26.5 |  | 67R Tanapox host range protein |
| 224 | 42 | F | 232a | 92 | T | fpv217 | 328 | 0 | 24.1 | 9.5 |  | Hypothetical protein |
| - | - | - | 232b | 210 | T | fpv217 |  | 15.7 | 55.5 | 9.5 |  |  |
| 225a | 37 | T | 233 | 474 | I | fpv218 | 461 | 6.5 | 77.0 | 6.3 |  | Ankyrin repeat family |
| 225b | 190 | T | - | - | - | fpv218 | 461 | 32.0 | - | 29.9 |  | Ankyrin repeat family |
| 225c | 108 | T | - | - | - | fpv218 | 461 | 32.9 | - | 28.6 |  | Ankyrin repeat family |
| **226** | **440** | **I** | **234** | **441** | **I** | **fpv219** | **434** | **93.2** | **83.7** | **84.2** |  | **Ankyrin repeat family** |
| 227 | 183 | I | 235 | 183 | I | fpv221 | 183 | 94.0 | 88.5 | 88.5 | A47L | A47L homolog |
| **228** | **747** | **I** | **236** | **747** | **I** | **fpv222** | **747** | **92.9** | **83.4** | **82.9** |  | **Ankyrin repeat family** |
| 229 | 44 | F | 237 | 69 | F | fpv223 | 141 | 41.4 | 33.3 | 22.0 |  | Ankyrin repeat family |
| 230 | 106 | F | - | - | a | cnpv298/fpv223-225 | 571 | - | - | 8.58 |  | Ankyrin repeat family |
| - | - | a | 238 | 59 | F | fpv224 | 146 | - | 32.9 | - |  | Ankyrin repeat family |
| - | - | a | 239 | 61 | F | fpv225 | 104 | - | 45.7 | - |  | Vaccinia B20R homolog |
| **231** | **293** | **I** | **240** | **293** | **I** | **fpv226** | **292** | **94.5** | **88.4** | **88.7** | **B1R** | **Serine/threonine protein kinase** |
| **232** | **361** | **I** | **241** | **360** | **I** | **fpv227** | **361** | **95.0** | **86.2** | **88.9** |  | **Ankyrin repeat family** |
| 233a | 83 | F | 242 | 48 | F | fpv228 | 525 | 5.6 | 5.1 | 12.4 |  | Ankyrin repeat family |
| 233b | 39 | F | - | - | - | fpv228 | 525 | 10.0 | 6.1 | 5.3 |  | Ankyrin repeat family |
| 233c | 101 | F | - | - | - | fpv228 | 525 | 6.3 | 6.1 | 14.3 |  | Ankyrin repeat family |
| 234 | 319 | F | 243 | 319 | F | Orientia tsutsugamushi str. Boryong/303 | 500 | 92.5 | 22.2 | 21.4 |  |  |
| 235 | 503 | I | 244 | 505 | I | cnpv301/fpv233 | 527 | 92.7 | 36.6 | 37.1 |  | Ankyrin repeat family |
| - | - | a | 245 | 75 | F | CNPV022/fpv027 | 358 | - | 8.5 | - |  | Serpin family |
| 236 | 185 | I | 246 | 182 | I | fpv229 | 180 | 92.4 | 77.8 | 80.0 |  | Hypothetical A47L-like protein |
| 237a | 51 | F | 247 | 514 | I | cnpv303/fpv230-231 | 256 | 6.7 | 47.8 | 2.5 |  | Ankyrin repeat family |
| 237b | 140 | F |  |  |  | cnpv303/fpv230-231 | 256 | 24.5 | - | 15.0 |  | Ankyrin repeat family |
| 237c | 132 | F |  |  |  | cnpv303/fpv230-231 | 256 | 23.1 | - | 11.2 |  | Ankyrin repeat family |
| **238** | **481** | **I** | **248** | **481** | **I** | **fpv232** | **482** | **93.8** | **86.3** | **85.7** |  | **Ankyrin repeat family** |
| **239** | **502** | **I** | **249** | **414** | **I** | **fpv233** | **512** | **76.5** | **69.7** | **85.6** |  | **Ankyrin repeat family** |
| 240a | 80 | F | 250 | 422 | I | fpv234 | 428 | 16.4 | 80.4 | 15.9 |  | Ankyrin repeat family |
| 240b | 57 | F | - | - | - | fpv234 | 428 | 11.4 | - | 9.8 |  | Ankyrin repeat family |
| 240c | 41 | F | - | - | - | fpv234 | 428 | 5.9 | - | 7.0 |  | Ankyrin repeat family |
| - | - | a | 251 | 148 | I | fpv235 | 143 | - | 65.5 | - |  | C-type lectin family |
| **241** | **279** | **I** | **252** | **274** | **I** | **fpv236** | **280** | **87.8** | **77.7** | **76.8** |  | **N1R/p28 family** |
| 242 | 122 | I | 253 | 70 | I | fpv237 | 67 | 45.6 | 57.1 | 26.8 |  | Hypothetical protein |
| 243 | 72 | F | 254 | 163 | I | fpv239 | 163 | 34.4 | 79.1 | 31.9 | A40R | C-type lectin family |
| **244** | **411** | **I** | **255** | **411** | **I** | **fpv240** | **410** | **88.8** | **78.8** | **79.1** |  | **Ankyrin repeat family** |
| - | - | a | 256 | 109 | F | GTPV_gp138 | 634 | - | 5.1 | - |  | Hypothetical protein |
| - | - | a | 257 | 185 | I | fpv241 | 186 | - | 48.4 | - |  | Ankyrin repeat family |
| 245 | 209 | I | 258 | 210 | I | cnpv313 | 218 | 92.4 | 51.6 | 51.14 |  | Ig domain protein |
| 246 | 628 | I | 259 | 643 | I | cnpv314/fpv242-243 | 584 | 92.4 | 50.4 | 50.9 |  | Ankyrin repeat family |
| 247 | 192 | I | 260 | 192 | I | Cnpv309/fpv241 | 196 | 91.7 | 29.8 | 29.8 |  | Ankyrin repeat family |
| - | - | a | 261 | 53 | F | cnpv014/fpv017 | 490 | - | 5.5 | - |  |  |
| 248 | 54 | F | 262 | 58 | F | cnpv320/fpv017 | 469 | 65.6 | 5.1 | 4.3 |  | Ig domain protein |
| 249 | 667 | I | 263a | 366 | T | fpv244 | 688 | 48.3 | 42.8 | 79.2 |  |  |
| - | - | - | 263b | 246 | T | fpv244 | 592 | 34.6 | 29.8 |  |  | Ankyrin repeat family |
| 250 | 75 | F | 264 | 440 | I | fpv245 | 463 | 12.9 | 76.8 | 10.7 |  | Ankyrin repeat family |
| 251a | 227 | T | 265a | 274 | T | cnpv014/fpv017 | 490 | 65.7 | 27.9 | 27.1 |  | Ig Domain protein |
| 251b | 235 | T | 265b | 235 | T | cnpv014/fpv017 | 490 | 75.4 | 25.1 | 28.0 |  | Ig Domain protein |
| 252 | 585 | I | 266 | 584 | I | fpv246 | 592 | 94.5 | 85.1 | 85.3 |  | Ankyrin repeat family |
| 253 | 122 | I | 267 | 124 | I | fpv247 | 124 | 91.9 | 83.0 | 82.3 |  | Efc family |
| 254 | 149 | I | 268 | 149 | I | fpv248 | 151 | 92.0 | 79.5 | 78.2 |  | N1R/p28 family |
| - | - | a | 269 | 628 | I | fpv162 | 603 | - | 41.3 | - |  | Ankyrin repeat family |
| 255 | 107 | I | 270 | 52 | F | fpv249 | 105 | 38.3 | 31.1 | 61.4 |  | Hypothetical protein |
| 256 | 124 | I | 271 | 124 | I | fpv258 | 123 | 82.3 | 67.2 | 68.0 | A40R | C-type lectin family |
| - | - | a | 272 | 122 | I | fpv256 | 122 | - | 78.7 | - |  | C-type lectin family |
| - | - | a | 273 | 467 | I | cnpv320/fpv017 | 469 | - | 82.7 | - |  |  |
| - | - | a | 274 | 46 | F | cnpv006/fpv002.5 | 182 | - | 14.3 | - |  |  |
| **257** | **222** | **I** | **275** | **222** | **I** | **fpv259** | **222** | **98.2** | **91.4** | **91.4** |  | **Hypothetical protein** |
| 258 | 66 | F | - | - | a | Aasi_1435/ Cnpv021 | 1585 | - | - | 2.3 |  | Hypothetical protein Aasi_1435 [Candidatus Amoebophilus asiaticus 5a2] |
| - | - | a | 276 | 115 | F | cnpv310/fpv246 | 537 | - | 8.7 | - |  |  |
| - | - | a | 277 | 164 | F | cnpv319/fpv246 | 739 | - | 8.3 | - |  |  |
| **259** | **200** | **I** | **278** | **204** | **I** | **fpv260** | **205** | **87.8** | **77.7** | **76.6** | **A40R** | **C-type lectin family** |

***Bold and Italic:*** The 90 core genes conserved in all ChPVs which are involved in essential functions such as replication, transcription and virion assembly

**Bold:** An additional 88 genes conserved in all avipoxviruses

Gene status is depicted as “I” for intact genes, “F” for fragmented genes (disrupted at 5’ end; not expected to be functional), “T” for truncated genes (intact 5’ end but <80% length of orthologue), “E” for genes than are longer than their orthologue and “a” where the gene is absent from the genome.

^α^ best BlastP hit (Where the best BlastP hit was a canarypox virus gene (CNPV), the homologous fowlpox virus gene is indicated where applicable. Some CNPV genes are not present in FWPV.
