# Supplementary figures and images for: The complete genome sequences of poxviruses isolated from a penguin and a pigeon in South Africa and comparison to other sequenced avipoxviruses
Source: BMC Genomics. 2014 Jun 12;15:463. doi: 10.1186/1471-2164-15-463 (PMC4229897; doi:10.1186/1471-2164-15-463)

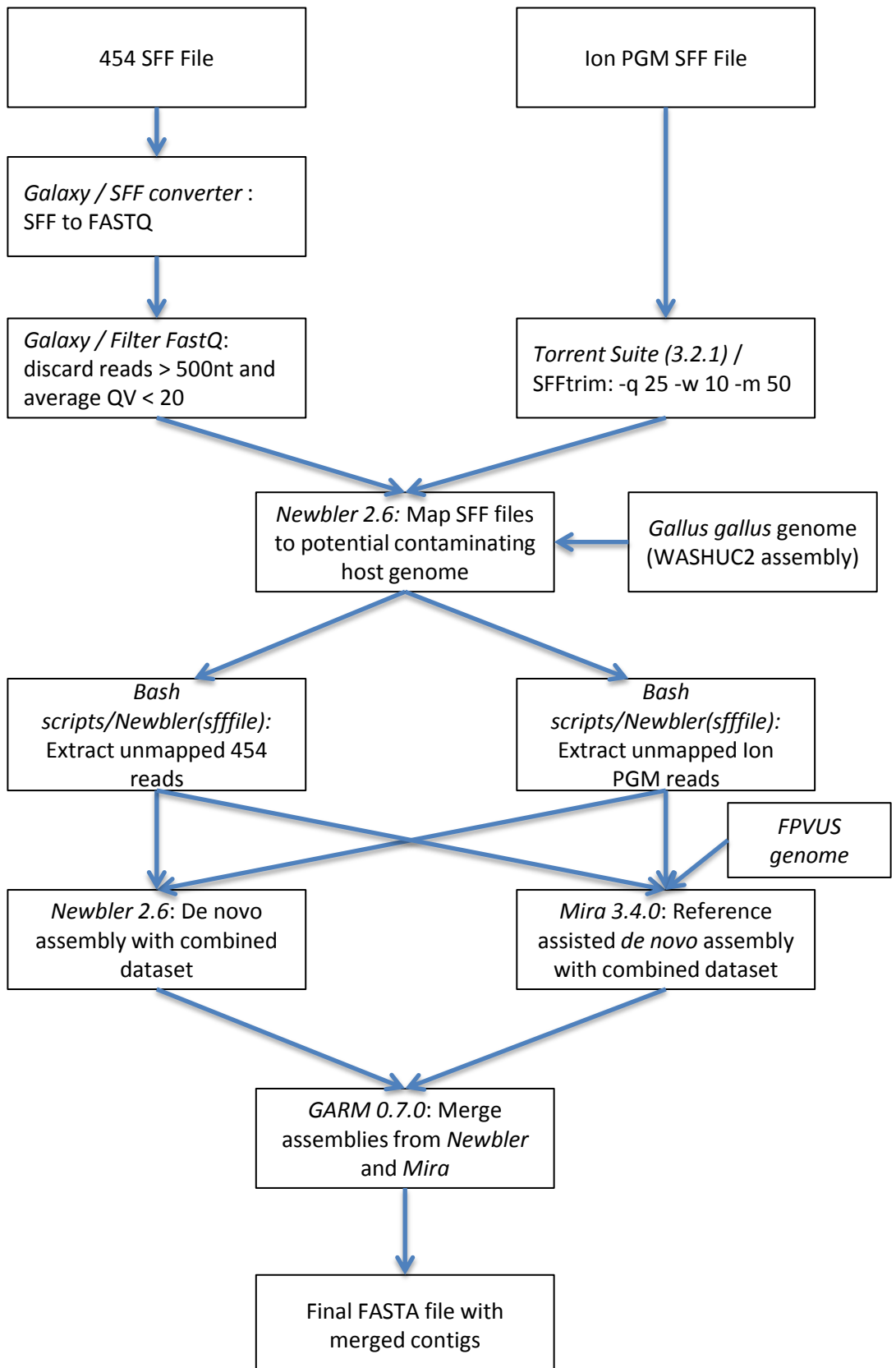

Supplement: Additional file 2 — Flow diagram depicting the customized bioinformatics pipeline used to analyse FeP2 and PEPV sequencing data. [file 1471-2164-15-463-S2.pdf]
